# CSF Proteomics of Patients with Hydrocephalus and Subarachnoid Haemorrhage

**DOI:** 10.1515/tnsci-2019-0040

**Published:** 2019-10-02

**Authors:** Bartosz Sokół, Bartosz Urbaniak, Bartosz Zaremba, Norbert Wąsik, Zenon J. Kokot, Roman Jankowski

**Affiliations:** 1 Department of Neurosurgery, Poznan University of Medical Sciences. Ul. Przybyszewskiego 49, 60-355 Poznan, Poland; 2 Department of Inorganic and Analytical Chemistry (Faculty of Pharmacy), Poznan University of Medical Sciences. Ul. Grunwaldzka 6, 60-780 Poznan, Poland

**Keywords:** subarachnoid haemorrhage, outcome prediction, biomarkers, mass spectrometry

## Abstract

**Background:**

The pathophysiology of brain injury following aneurysmal subarachnoid haemorrhage (SAH) is associated with numerous mediators. The aim of the study is to analyse protein changes after SAH in cerebrospinal fluid (CSF) using mass spectrometry (MS).

**Methods:**

CSF samples were obtained from forty-four control subjects, seven good outcome and ten poor outcome SAH patients. CSF samples were collected at specific time intervals after SAH (days 1, 5 and 10). MALDI-TOF (Matrix Assisted Laser Desorption/Ionization Time-of-Flight) and ClinProTools software were utilised for MS, MS/MS (Mass Spectrometry) spectra collection and analysis. Selected masses were identified. The MALDI-TOF profiling experiments allowed for the targeted selection of potential markers in SAH. The study was performed in three steps by comparison of CSF samples: (1) from the control group and SAH patients (both good and poor outcome groups); (2) collected on days 1, 5 and 10 within the groups of poor SAH and good SAH patients, respectively; (3) from poor outcome SAH and good outcome patients at days 1, 5 and 10.

**Results:**

15 new proteins whose CSF level is alternated by SAH presence, SAH treatment outcome and time passed since aneurysm rupture were identified.

**Conclusions:**

We demonstrated new proteins which might play a role in different stages of subarachnoid haemorrhage and could be a new target for further investigation.

## Introduction

The global incidence of aneurysmal subarachnoid haemorrhage (SAH) is 6.67 per 100,000 persons, and nearly half a million individuals suffer from SAH each year [1]. Approximately 15% will die before reaching the hospital, 25% die within 24 hours, and 45% die within 30 days. Only one-third will make a full recovery after treatment [2]. SAH is associated with a variety of pathological changes affecting both glial and neuronal brain cells. In patients with SAH, neurological dysfunction presents within several hours of injury. This decline was initially attributed to the progressive mass effect secondary to acute SAH; however, current evidence suggests that the progression to cerebral ischaemia is far more complex [3]. Interest has grown around early brain injury (EBI), which by definition covers all the pathophysiological processes occurring in the first 72 hours, including cortical spreading depression, microvascular injury, blood-brain barrier disruption, neuroinflammation and global cerebral oedema [4]. Recent evidence points to neuroinflammation as a key mediator of injury expansion and behavioural deficits [5]. The underlying mechanisms contributing to injury expansion following SAH are poorly understood, thereby limiting the number of effective pharmaceutical treatment options [6, 7, 8]. Changes in protein expression may provide more meaningful information regarding the complex mechanisms underlying neurological injury following SAH. Over the past several decades, a variety of proteins have been tested as therapeutic targets [9]. Unfortunately, despite showing great promise in the course of preclinical trials, most experimental drugs fail to lead to significant improvement in the outcome of patients with SAH [6, 9, 10, 10, 11, 12]. Thus far, the only medication which has been shown to reduce cerebral infarction and improve outcome after SAH is nimodipine [13]. Therefore, a new approach is required to investigate the pathophysiology at the molecular and cellular levels in the hope of finding novel therapeutic targets [4, 14]. This paper is a preliminary study performed on a small number of patients to demonstrate presence of new cerebrospinal fluid (CSF) biomarkers representing different stages of SAH by the use of matrix-assisted laser desorption/ionisation time-of-flight mass spectrometry (MALDI-TOF). This paper should demonstrate the role of other proteins not previously related to SAH and encourage further investigations with less sophisticated methods.

## Methods

### Ethics and Consent

This is a prospective observational study conducted at a single medical centre in accordance with the Declaration of Helsinki. The study was approved by the Local Bioethics Committee (approval no 983/13). The Local Bioethics Committee approved the study protocol, consenting protocol and consent forms. Either the patient or the next of kin gave consent for entry to the study, and the use of blinded medical data for analysis and publication.

### Management, Definitions, End points

On admission, the clinical status was assessed using GCS and specific SAH-grading scales (Hunt & Hess [HH] [15], World Federation of Neurosurgical Societies [WFNS] [16]). An initial head CT scan was used to confirm SAH and assess the presence of acute hydrocephalus. EVD was placed following endovascular treatment in patients with GCS score below 15 and radiological markers of hydrocephalus. A second CT scan was performed within 24 hours of aneurysm occlusion and EVD placement to assess any procedure related to brain injury. Patients received a continuous infusion of nimodipine for at least ten days; hypotension was avoided using vasopressors, and euvolemia was maintained. EVD infection screening involved CSF cell count at least twice per patient, and CSF culture at least once on day ten post-SAH. The primary endpoint was the treatment outcome assessed at three months using the Glasgow Outcome Scale (GOS) [17]. Patients were divided into two groups according to GOS. Good outcome (GO-SAH) consisted of those with no disability, moderate disability and severe disability (GOS grade 5, 4 and 3); poor outcome (PO-SAH) were those with persistent vegetative state or death (GOS grade 2 and 1). We decided to divide patients in such outcome fashion as we wanted to demonstrate the most extreme values in poor outcome treatment. Delayed cerebral ischaemia (DCI) related infarction was defined as a new cerebral infarction identified on a head CT scan within six weeks of rupture and not present on the immediate post-treatment scan (as proposed by Vergouwen [18]).

### Population, Inclusion and Exclusion Criteria

**Figure 1:**
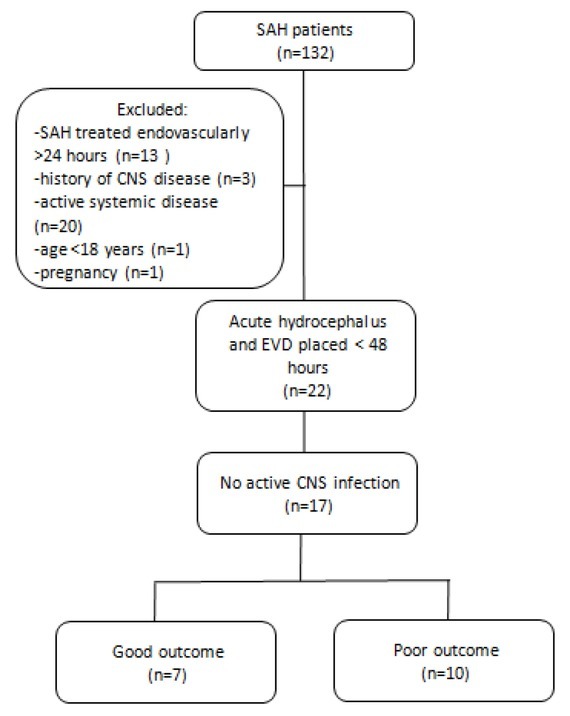
Study recruitment flowchart.

The analysed group consisted of 17 SAH patients (9 males and 8 females, mean age 60,9 years). There were seven good outcome patients (3 males and 4 females, mean age 60,42 years) and ten poor outcome patients (6 males and 4 females, mean age 62 years). Control group consisted of 44 subjects with mean age 56,1 years. CSF samples were obtained during spinal anaesthesia from age and sex-matched patients (p = 0.56 and p = 0.68, respectively) with a negative history of CNS disease. There were 5 patients with the aneurysm located at the middle cerebral artery (1 in good outcome and 4 in poor outcome patients), 7 patients had aneurysm located at the anterior communicating artery (3 in good outcome and 4 in poor outcome patients), 3 patients had aneurysm at the anterior cerebral artery, 2 patients with good and 1 patient with poor outcome. One patient with good outcome had aneurysm located at the basilar artery and one patient with poor outcome had aneurysm located at the posterior cerebral artery. Average aneurysm size was 4,9 mm, in the good outcome it was 4,6 mm and in the poor outcome it was 5 mm. Details are demonstrated in table 1. Inclusion criteria were as follows: (1) SAH treated endovascularly within 24 hours of rupture, (2) external ventricular drainage (EVD) placed within 48 hours. Exclusion criteria for the study were: (1) history of CNS disease, (2) active CNS infection, (3) active systemic disease (diabetes mellitus, rheumatoid arthritis, malignancy, cirrhosis, renal failure), (4) age below 18, (5) pregnancy. Patients recruitment flowchart is demonstrated in figure 1. Details demonstrating patients severity of the disease on admission and treatment outcome are presented in table 2. Mean value of Fisher CT scale in poor outcome patients group was four points and in good outcome patients was four points. Mean value for the modified Fisher scale was four points in poor outcome patients and two points in good outcome patients. There was a statistically significant difference p=0.011. 60% of patients with poor outcome and 29% with good outcome had intracerebral hematoma confirmed on CT. 100% of poor outcome patients and 57% good outcome patients had ventricular blood on CT and there was a statistical significance p=0.023. 80% of poor outcome and 42% of good outcome patients had cerebral infarction due to DCI and there was no significant difference. WFNS scale on admission was five in poor outcome and four in a good outcome. Hunt and Hess scale on admission was five in poor outcome and three in good outcome patients. GCS on admission in poor outcome patients was five and ten in good outcome patients. GOS at three

**Table 1:** Study group patients` characteristic.

Variables:	Descriptive statistics			
	SAH patients n = 17	Good outcome SAH patients n=7	Poor outcome SAH patients n=10	Control group n=44
**Male**	9 (53%)	3 (42%)	6 (60%)	20 (45%)
**Age (years)**	60,9 ± 12,2	60,42±15,2	62±10,5	56,1 ± 14,2
**Aneurysm location:**				
**Middle cerebral artery**	5 (29%)	1	4	
**Anterior communicating artery**	7 (41%)	3	4	
**Anterior cerebral artery**	3 (18%)	2	1	
**Basilar artery**	1 (6%)	1	-	
**Posterior cerebral artery**	1 (6%)	-	1	
**Aneurysmal size (mm)**	4,9 ± 1,9	4,6±1.95	5±1,9	

Values are presented as: (1) mean ± standard deviation for continuous data; (2) count (percentage) for categorical/ordinal data.

**Table 2:** Details demonstrating the severity of disease and treatment outcome.

	PO patients	GO patients	p-value
Fisher CT scale	4[4;4]	4 [2;4]	0.097
modified Fisher scale	4[3;4]	2[1;3]	**0.011**
ICH on CT	60%	29%	0.201
Ventricular blood CT	100%	57%	**0.023**
Cerebral infarction due to DCI	80%	42%	0.115
WFNS on admission	5[4;5]	4[2;5]	0.070
H&H on admission	5[4;5]	3[2;5]	0.053
GCS on admission	5[4;9]	10[5;13]	0.183
GOS at three months	1[1;2]	5[3;5]	**0.0004**

Values are presented as: (1) mean ± standard deviation for continuous data; (2) median [lower quartile, upper quartile] for ordinal data; (3), the percentage for categorical data. Abbreviations: WFNS = World Federation of Neurosurgical Societies scale; HH = Hunt and Hess scale; GCS = Glasgow Coma Scale; GOS = Glasgow outcome scale.

months was one in poor outcome patients and five in good outcome patients and there was a statistical significance p=0.0004.

### CSF Sample Collection

CSF samples were collected from the EVD at three-time points, on the 1st, 5th and 10th day after SAH. The CSF samples were centrifuged at the room temperature for 15 minutes with 2000 g-force applied, and the supernatants were transferred to fresh tubes and stored at -81°C until MALDI-TOF analysis was carried out. CSF samples were stored at -81°C for approximately 24 months.

### Sample processing, MALDI-TOF analysis and profiling studies

The Bradford method was used for the determination of total protein level in all analysed samples of CSF. The enrichment and separation procedure for purifying peptides or low molecular weight proteins was performed using reversed phase ZipTip (Millipore, C18, 10 μL) pipets tips. Samples were processed by ZipTip according to the following protocol: equilibrating and washing of the tip-end by acetonitrile (ACN) and 0.1% TFA; sample purification and enrichment (10 cycles); elution using ACN and 0.1% TFA (50:50,v/v%). The elution mixtures were mixed with the matrix (HCCA, alpha-cyano-4-hydroxycinnamic acid) in the ratio of 1:10 (eluate: HCCA, v/v%) and spotted (in a replication of three) on the target plate (AnchorChip Standard 800 mm, Bruker, Germany). The mass spectrometry experiments were performed using MALDI-TOF apparatus (UltrafleXtreme, Bruker), equipped with the FlexControl and FlexAnalysis modules, allowing for data acquisition and data/spectra treatment. The ClinProt standard was used as a mass standard. Spectra were collected as a sum of a total of 2500 laser shots, while a single laser beam was complexed from 500 light pulses and 1000 Hz of frequency. MS spectra were collected at a mass range of 1-10 kDa. The detailed sample processing procedure is attached in Supplementary Material 1.

### Analyzed groups

Statistical analysis of experimental MS data was divided and performed in three separate steps:

(1) comparison of CSF samples derived from the control group and SAH patients (control vs GO-SAH + PO-SAH; control vs GO-SAH; control vs PO-SAH); (2) comparison of CSF samples from days 1, 5 and 10 within the GO-SAH group, and also within the PO-SAH group; (3) comparison of CSF samples derived from GO-SAH and PO-SAH patients on days 1, 5 and 10.

## Data analysis - the statistical evaluation

The ClinProTools software (version 3.0, Bruker Daltonics) was used for the assessment of the statistical parameters of MS spectra obtained during profiling experiments. All spectra (1-10 kDa range) were normalised and processed with the following schedule: normalisation to the total ion current (TIC), recalibration using the prominent common m/z values, baseline “top hat” subtraction, minimum baseline width 10%, smoothing, signal-to-noise ratio (S/N) threshold 5, peak picking and peak calculation operation. A total average spectrum was calculated from the preprocessed spectra. In all tests p<0.05 was considered to indicate statistical significance. The peak statistic was described by the following parameters: DAve- difference between the maximal and minimal average peak intensity of all classes; PTTA (p-value for normally distributed data); PWKW (p-value for not normally distributed data); PAD (p-value of the Anderson-Darling test gives information about data distribution profile- normal/not normal). For further statistical evaluation, the three different algorithms were used to obtain the discriminating model: genetic algorithm (GA), quick classifier (QS) and supervised neural network (SNN). Each algorithm indicated a combination of the differentiating peaks (m/z, Da) along with specific range of masses where selected peak could have appeared (Supplementary Material 1,2,3).

### MALDI-TOF analysis - mass identification procedure

The mass identification was made excluding the digestion protocol. Skipping of trypsin digestion procedure allowed for analysis of CSF samples in which the low molecular proteins and polypeptides or its fragments were intact as described above in step first (Sample processing, MALDI-TOF analysis and profiling studies). Otherwise, application proteolytic enzymes (digestion protocol) would completely change the image of CSF profile, and making impossible the mutual comparison of masses obtained during profiling of intact low molecular proteins and polypeptides with those obtained during the identification procedure. The detailed mass identification procedure is given in Supplementary Material 1. MS spectra were acquired in the mass range 700-3500 Da. Fixed laser intensity and 2500 shots per spectrum were used. The Peptide Calibration Standard II (Bruker, Germany) was used as a mass standard. Before each MS-analysis, the apparatus was precalibrated according to the reference masses, and then, based on the obtained MS mass list, the MS/MS mode was applied. Identification of the proteins was performed by the Mascot platform using the SwissProt database. The following protein search parameters were set: precursor ion mass tolerance ±50 ppm, fragment ion mass tolerance ±0.7 Da, peptide mass charge +1.

## Results

### Proteomic findings

The MALDI-TOF profiling experiments of CSF samples derived from SAH patients, and the application of advanced statistical analysis allowed for the targeted selection of proteins related to the treatment outcome in SAH patients. There were no statistical differences between the control and study groups about age and sex (p = 0.56 and p = 0.68, respectively). The statistical analysis of the mass profile of the CSF samples revealed a set of masses, whose occurrence and levels varied with the analysed group (GO-SAH, PO-SAH and controls), and day of collection. It allowed to verify and identify the detected peaks referring to the particular masses. We identified a set of masses of low molecular weight proteins and polypeptides.

### SAH patients vs control group

**Figure 2:**
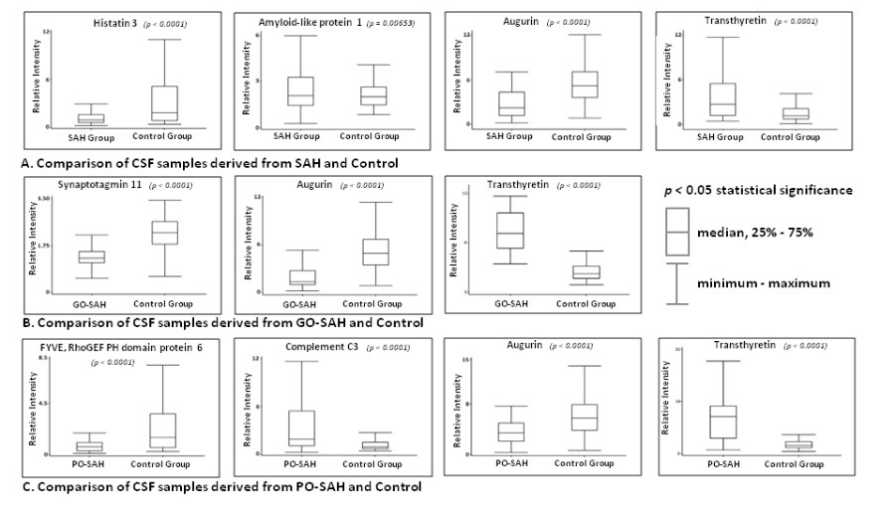
CSF proteins of significantly different level between the healthy control group and subarachnoid haemorrhage patients. (A) CSF proteins of significantly different level between the healthy control group and SAH patients (both GO-SAH and PO-SAH); (B) CSF proteins of significantly different level between the healthy control group and GO-SAH patients; (C) CSF proteins of significantly different level between the healthy control group and PO-SAH patients. All CSF samples (1st, 5th and 10th day after SAH) were included in comparisons. Abbreviations: GO-SAH = good outcome; PO-SAH = poor outcome, SAH = subarachnoid haemorrhage

CSF proteins showing significantly different levels between SAH patients and the control group are amyloid like protein, transthyretin, augurin, and histatin 3 protein (Table 3). The levels of amyloid like protein and transthyretin were increased in the SAH patients, whilst those of augurin and histatin 3 were decreased (Figure 2).

**Table 3:** Protein expression between different groups of SAH patients and the control group in relation to treatment outcome.

Identified protein	m/z [Da]	p-value	Protein levels (Mean ± SD)	
**SAH PATIENTS AND CONTROL GROUP**			**SAH Group (n=17)**	**Control Group (n=44)**
**Amyloid-like protein 1**	2342.65	0.00653	3.01 ± 2.38	2.30 ± 1.04
**Augurin**	2985.45	< 0.0001	3.14 ± 2.59	5.88 ± 2.92
**Histatin 3**	1283.21	< 0.0001	1.49 ± 1.58	3.48 ± 3.62
**Transthyretin**	3444.24	< 0.0001	10.20 ± 9.27	3.10 ± 2.29
**GOOD OUTCOME SAH PATIENTS AND CONTROL GROUP**			**GO-SAH (n=7)**	**Control Group (n=44)**
**Augurin**	2985.45	< 0.0001	2.17 ± 1.70	5.91 ± 2.90
**Synaptotagmin 11**	1915.64	< 0.0001	1.46 ± 0.55	2.50 ± 0.91
**Transthyretin**	3444.24	< 0.0001	10.37 ± 10.65	3.11 ± 2.29
**POOR OUTCOME SAH PATIENTS AND CONTROL GROUP**			**PO-SAH (n=10)**	**Control Group (n=44)**
**Augurin**	2985.45	< 0.0001	3.61 ± 2.99	5.83 ± 2.94
**Complement C3**	1866.42	< 0.0001	4.52 ± 5.92	1.33 ± 1.41
**FYVE, RhoGEF PH domain protein** 6	1104.80	< 0.0001	0.84 ± 0.74	2.45 ± 2.51
**Transthyretin**	3444.24	< 0.0001	10.74 ± 7.22	3.08 ± 2.29

Abbreviations: m/z - mass (m) to charge (z) ratio, Da – Daltons, p-value – probability value, in all tests p<0.05 was considered to indicate statistical significance (PTTA test for normally distributed data and PWKW test for not-normally distributed data).

### Good outcome SAH patients vs. control group

**Figure 3:**
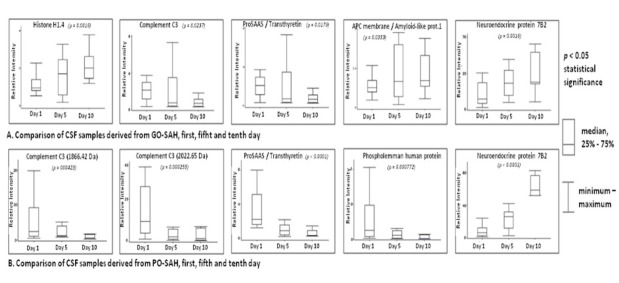
CSF proteins of significantly different level derived from poor and good treatment outcome SAH groups collected at the constant time interval 1st, 5th and 10th day after bleeding. (A) CSF proteins of significantly different level derived from GO-SAH patients collected at the constant time interval 1st, 5th and 10th day after bleeding; (B) CSF proteins of significantly different level derived from PO-SAH patients collected at the constant time interval 1st, 5th and 10th day after bleeding.

Comparison of CSF samples of GO-SAH patients with the control group showed significant differences for: augurin, synaptotagmin-11 protein and transthyretin (Table 3). The transthyretin level was increased in the GO-SAH group, whilst the levels of augurin and synaptotagmin-11 were decreased (Figure 3).

### Poor outcome SAH patients vs. control group

When samples from the PO-SAH group were compared with the controls, the following peaks were identified: augurin, complement C3, FYVE, RhoGEF and PH Domain containing protein 6 and transthyretin. Levels of augurin and FYVE, RhoGEF and PH domain protein 6 were decreased in PO-SAH and levels of complement C3 protein and transthyretin were increased in PO-SAH (Table 3, Figure 2). Detailed statistical data are given in ‘Supplementary Material 2’.

### Protein expression in good and poor outcome SAH patients on days 1,5 and 10 post-SAH

Statistical comparison of CSF samples from good and poor outcome patients collected on days 1, 5 and 10 post-SAH led to the selection of a set of proteins which could represent markers for progress and recovery (Table 4, Figure 3).

**Table 4:** Proteins expression during treatment on day one, fifth and tenth in good and poor outcome SAH patients.

Identified protein	m/z [Da]	p-value		Protein levels (Mean ± SD)	
**GOOD OUTCOME SAH PATIENTS (n=7)**			**Day 1**	**Day 5**	**Day 10**
**Histone H1.4**	1929.51	0.0016	1.21 ± 0.47	2.18 ± 1.83	2.37 ± 0.96
**Complement C3**	2022.65	0.0237	10.71 ± 10.93	11.72 ± 17.7	2.75 ± 2.60
**ProSAAS protein (2045.03 Da) / Transthyretin (2040.52)**	2044.52	0.0179	2.40 ± 1.83	2.14 ± 2.45	0.85 ± 0.55
**APC membrane recruitment (2350.20 Da) / Amyloid like protein (2342.18 Da)**	2360.20	0.0353	2.05 ± 0.87	3.25 ± 2.62	3.85 ± 3.35
**Neuroendocrine protein 7B2**	3516.13	0.0016	15.26 ± 12.75	28.42 ± 15.71	37.72 ± 20.91
**POOR OUTCOME SAH PATIENTS (n=10)**			**Day 1**	**Day 5**	**Day 10**
**Complement C3**	1866.42	0.000423	6.56 ± 7.15	2.46 ± 1.73	0.75 ± 0.66
**Complement C3**	2022.65	0.000255	19.81 ± 15.59	13.71 ± 25.78	3.04 ± 3.74
**ProSAAS protein (2045.03 Da) / Transthyretin (2040.52)**	2044.52	0.000008	3.73 ± 2.50	3.69 ± 7.04	0.55 ± 0.44
**Phospholemman human protein**	2755.26	0.000772	36.35 ± 40.58	16.38 ± 21.07	2.78 ± 3.57
**Neuroendocrine protein 7B2**	3516.13	0.0000354	8.31 ± 6.30	25.14 ± 14.68	56.62 ± 32.57

Abbreviations: m/z - mass (m) to charge (z) ratio, Da – Daltons, p-value – probability value, in all tests p<0.05 was considered to indicate statistical significance (PTTA test for normally distributed data and PWKW test for not-normally distributed data).

### Good outcome SAH patients on days 1, 5 and 10 post SAH

In the good outcome group, the peaks represented histone H1.4, complement component C3, proSAAS protein and transthyretin, (within one mass), APC membrane recruitment protein and amyloid like protein (within one mass), and neuroendocrine protein 7B2. Where two different proteins appear within one mass range, further investigation will be required to establish whether one or both may be the potential marker. In the good outcome patients elevation of histone H1.4, APC membrane recruitment protein/amyloid like protein and neuroendocrine Protein 7B2 was demonstrated, whilst complement C3, proSAAS protein/transthyretin were reduced (Table 4, Figure 3).

### Poor outcome SAH patients on days 1, 5 and 10 post SAH

In the poor outcome group the peaks represented complement component C3 (at two points), proSAAS protein and transthyretin (within one mass), phospholemman human protein and neuroendocrine protein. In the poor outcome group neuroendocrine Protein 7B2 was elevated, whilst complement C3, proSAAS protein/transthyretin and phospholemman human protein levels were decreased (Table 4, Figure 3). Detailed statistical data are given in ‘Supplementary Material 3’.

### Comparison between good and poor outcome SAH patients on days 1, 5, 10

**Figure 4:**
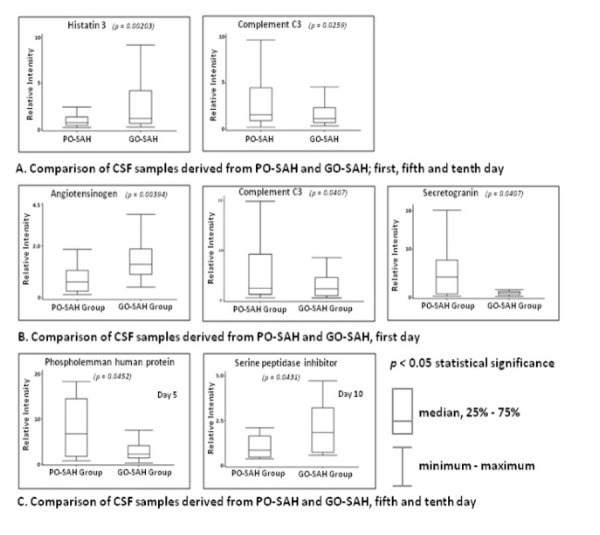
CSF proteins of the significantly different level compared between GO-SAH and PO-SAH patients on day 1st, 5th and 10th post SAH. (A) CSF proteins of the significantly different level compared between GO-SAH and PO-SAH patients on day 1st; (B) CSF proteins of the significantly different level compared between GO-SAH and PO-SAH patients on day 5th; (C) CSF proteins of the significantly different level compared between GO-SAH and PO-SAH patients on day 10th.

Comparison of samples taken on all three collection days between the good and poor outcome patients identified complement C3 and histatin 3 (Table 5); the former was increased in the poor outcome group, whilst the latter was reduced (Figure 4).

**Table 5:** Proteins expression difference between poor and good outcome SAH patients during treatment.

Identified protein	m/z [Da]	p-value	Protein levels (Mean ± SD)	
**GOOD OUTCOME SAH AND POOR OUTCOME SAH DAY**	**1, 5 and 10 (n=41)**		**PO-SAH (n=20)**	**GO-SAH (n=21)**
**Complement C3**	1866.42	0.0259	4.18 ± 5.68	2.00 ± 1.86
**Histatin 3**	1332.65	0.00203	1.29 ± 1.34	3.24 ± 3.63
**GOOD OUTCOME SAH AND POOR OUTCOME SAH DAY**	**1 (n=17)**		**PO-SAH (n=7)**	**GO-SAH (n=10)**
**Angiotensinogen**	1050.20	0.00394	1.07 ± 1.30	2.16 ± 1.35
**Complement C3**	1866.42	0.0407	6.57 ± 7.14	3.19 ± 2.70
**Secretogranin**	3201.05	0.0407	6.58 ± 7.31	1.46 ± 1.47
**GOOD OUTCOME SAH AND POOR OUTCOME SAH DAY**	**5 (n=14)**		**PO-SAH (n=7)**	**GO-SAH (n=7)**
**Phospholemman human protein**	2755.26	0.0452	16.38 ± 21.07	3.03 ± 2.26
**GOOD OUTCOME SAH AND POOR OUTCOME SAH DAY**	**10 (n=10)**		**PO-SAH (n=4)**	**GO-SAH (n=6)**
**Serine peptidase inhibitor**	1345.46	0.0431	1.03 ± 0.73	2.13 ± 1.36

Abbreviations: m/z - mass (m) to charge (z) ratio, Da – Daltons, p-value – probability value, in all tests p<0.05 was considered to indicate statistical significance (PTTA test for normally distributed data and PWKW test for not-normally distributed data).

### Comparison on day one

When the comparison was restricted to day 1, three proteins were identified: angiotensinogen, complement component C3 and secretogranin. Angiotensinogen was elevated in the good outcome patients and complement C3 and secretogranin were elevated in the poor outcome group (Table 5, Figure 4).

### Comparison on days 5 and 10

Similar analyses showed increased phospholemman human protein on day 5 in poor outcome patients and increased serine peptidase inhibitor protein on day 10 in good outcome patients (Table 5, Figure 4). Detailed statistical data are given in ‘Supplementary Material 3’.

## Discussion

There are many gaps in our understanding of the pathophysiology of SAH, and in particular of ways to predict and prevent the development of cerebral ischaemia [19]. Numerous groups, including our own, have embarked on the analysis of alterations of a variety of proteins in biological fluids with this aim in view. Our previous studies on sTLR 2 and 4 [20], sRAGE [21] and Clusterin [22] in CSF of SAH patients were limited by the methodology, i.e. ELISA assays of proteins preselected as a result of a thorough review of the stroke and neuroinflammatory disease literature. To overcome these limitations, we implemented proteomic techniques [23], since they allow the simultaneous, large-scale screening of all proteins in a biological sample [4]. In the current study, 15 entirely new potential targets deserving further investigation have been found. Three potential sources of such proteins need to be considered: (1) extravasated blood (with subsequent lysis of blood cells); (2) local production by neural tissue; (3) migration from the blood through a disrupted BBB. Below, we present a brief commentary on those proteins which we feel likely to be most important. Transthyretin (TTR) is the most widely described protein in the group. On a cellular level, it is harmful to cell survival following an ischaemic insult to the brain [24]. Patients suffering from an ischaemic stroke with an unfavourable outcome were found to have significantly lower TTR serum levels on admission [25]. On the contrary, TTR levels appear to be a negative predictor of poor outcome at three months following SAH [26]. In the present study, we found a significant increase in TTR levels in SAH patients compared with healthy controls, but no clear difference between good and poor outcome patients at any point. Histatin 3 forms part of the innate immune system and is currently thought that its main function is to limit infections in the oral cavity [27] . Histatin 3 inhibits NF-kB activation in both TLR4/MD2/CD14 and TLR2/CD14 mode [27]; no previous work has demonstrated its presence in CSF. In the current study, histatin 3 levels were significantly decreased in SAH patients, and also, the levels were significantly lower in the poor outcome group than in the good. Based on the available literature and our findings, we might speculate that CSF histatin 3 has a protective role in SAH pathophysiology. Augurin is a hormone-like protein that is thought to play a role in central nervous system (CNS) homeostasis [28, 29, 30, 31]. A decrease in augurin protein occurs at times when cells proliferate in the normal CNS injury response. The time-course of decreased augurin correlates with the acute and subacute response times to CNS injury as both gene and protein expression are decreased at 1-3 days post injury, but return to uninjured levels by day 7 [32]. Our study might suggest similar level trends. Augurin levels were significantly lower in SAH patients, but we found no significant difference between good and poor outcome patients, nor did we find significant changes with time. Synaptotagmin-11 regulates repair of injured astrocytes following acute brain injury [33] and also suppresses microglial activation under both physiological and pathological conditions, through the inhibition of cytokine secretion and phagocytosis [34]. In this study, synaptotagmin-11 was found to be decreased in good outcome patients compared with healthy controls, but there was no significant difference between the good and poor outcome group. This raises the possibility that synaptotagmin-11 may have a protective role. Complement C3 is an essential immune regulator of host defence against infection, cell integrity, and tissue homeostasis in the peripheral system. It is a significant mediator of cerebral injury following stroke [31]. Administration of a C3a-receptor antagonist resulted in commensurate neurological improvement and reduction of stroke volume in an animal model [35]. Complement C3 is produced by astroglia if a proinflammatory signalling pathway (involving NF-kB) is employed [36]. If levels for all three sampling days are compared, the levels are significantly greater in poor outcome patients than in good outcome patients and controls; if day one is taken separately, the good outcome patients had significantly lower levels than the poor outcome group. Another protein we found to be elevated was angiotensinogen; this is a moderately abundant glycoprotein which is the precursor to angiotensin peptides and is the only known naturally occurring renin substrate. It is also synthesized by astrocytes [37]. The rat angiotensinogen (rAT) gene, which codes for the protein precursor, is known to be rapidly induced in rat liver in the course of the acute phase response [38]. It was found to be significantly elevated in good outcome patients compared to the poor group on the first day post-SAH. Secretogranin is an acidic secretory protein in large dense-core vesicles of neural and neuroendocrine tissues [39]. It is an established ischaemia-induced gene product [40], but very little literature is available regarding its role. On day one post-SAH its level was significantly elevated in poor outcome patients. Complement C3, angiotensinogen and secretogranin levels in CSF are different between PO-SAH and GO-SAH groups on day one, but in overall comparison only Complement C3 differentiated between SAH and Control ( PO vs control). These findings might indicate that among those three potential early outcome biomarkers in SAH Complement C3 is the most promising one.

## Limitations

This research is clearly a preliminary study. (1) The numbers are small, and a single unusual reading may skew the results disproportionately. (2) Hydrocephalus itself aggravates brain injury and affects CSF protein changes, but it is prerequisite for the insertion of the EVD. (3) Despite the precautions taken, it is difficult to rule out the possibility of the infection completely. (4) Insertion of the drain alone may contribute to CSF changes. (5) It was necessary to use spinal CSF as a control. Since a very sensitive assay tool was being used, any of the above may have impacted the study results and separate analysis of each protein is required in the future.
