# Supplementary material for: CSF Proteomics of Patients with Hydrocephalus and Subarachnoid Haemorrhage
Source: Transl Neurosci. 2019 Oct 2;10:244–53. doi: 10.1515/tnsci-2019-0040 (PMC6778397; doi:10.1515/tnsci-2019-0040)
Supplement: Supplementary file 1 [file tnsci-10-244_sm.pdf]

SUPPLEMENTARY MATERIAL (SM) 1.

Manuscript title: **CSF PROTEOMICS OF PATIENTS WITH HYDROCEPHALUS AND SUBARACHNOID HAEMORRHAGE.**

Bartosz Sokół[1], Bartosz Urbaniak[2], Bartosz Zaremba[2], Norbert Wąsik[1], Zenon J. Kokot[2], Roman Jankowski[1].

1. Department of Neurosurgery, Poznan University of Medical Sciences.
2. Department of Inorganic and Analytical Chemistry, Poznan University of Medical Sciences.

Introduction to experimental part.

In the submitted manuscript the proteomic studies of cerebrospinal fluid (CSF) samples derived from SAH patients were presented. These studies were divided into two separate steps.

The first step of presented study was focused on profiling of intact low protein and poly-peptides in CSF samples using MALDI-TOF analysis, where all analyzed samples were treated with the ZipTip C18 procedure. Enriched samples were thereafter analyzed by MALDI-TOF MS in the range of 1-10 kDa. This step allowed for the collection of series of MS spectra (range 1-10 kDa) for all analyzed samples, both derived from SAH (good and poor outcome) as well as control, respectively. Afterwards, all of these collected spectra of intact low molecular prteins/polypeptides were analyzed by ClinnProTools program, whereby a series of characteristic and statistically important peaks were selected. Three different algorithms were used: genetic algorithm (GA), quick classifier (QC) and supervised neural network (SNN). All of these statistical algorithms returns back a series of statistically important peaks that can be potentially used as a markers of SAH. Moreover, some of selected peaks (masses, m/z) were common for more than one of these algorithms, what significantly increases the reliability of performed analysis. The drawback of this purpose is, that selected peaks are described only by mass (m/z Da) any of additional information (i.e. protein accession or sequence) is given.

Hence, in the second step of presented work, authors made an attempt to identify of the selected masses (m/z), using MALDI-TOF-TOF (MS/MS) technique coupled with nanoLC separation. As it was mentioned above, the profiling of intact low molecular proteins and polypeptides was performed in the first step and thus authors decided to omit the trypsine digestion protocol to make data obtained in these two steps comparable. The raw CSF samples (also enriched by ZipTip C18 procedure) were directly separated and fractionated by nanoLC procedure and further analyzed by MALDI-TOF-TOF MS/MS without treating them with proteolytic enzymes. Other words, authors did not involve the tryptic digestion procedure for this purpose. Skipping of trypsin digestion procedure allowed for analysis of CSF samples in which the the low molecular proteins and polypeptides or its

fragments were intact as described above in the step first. Otherwise, application proteolytic enzymes (digestion protocol) would completely change the image of CSF profile, and making impossible the mutual comparison of masses obtained in the step first and second.

Achieved MS/MS spectra were further treated with ProteinScape program basing on Mascot platform and using SwissProt database, and as a result the identification of some of selected masses (m/z) was possible. This approach allowed for the identification of almost fifteen proteins among all selected masses by ClinProTools. As a result of CSF samples profiling using ZipTip C18 procedure, MALDI-TOF MS and ClinProTools analysis and basing on calculation on various statistical algorithms the selection of characteristic masses was enabled. At every turn, statistic data obtained by ClinProTool gives back the mass (m/z) of the selected peaks along with specific range of masses where selected peak could have appeared. Thus, in a few cases, two of identified masses (m/z) that corresponds to two different proteins have been found in the same mass range. This situation was observed in the cases of ProSAAS proterin and transthyretin (selected mass 2044.52 Da, mass range 2038.94 – 2051.90 Da); and ACP membrane recruitment protein and amyloid like protein (selected mass 2342.18 Da, mass range 2336.23 – 2353.27 Da). For these reasons and taking into account that this may lead to wrong conclusions, authors strongly emphasized that in order to unambiguous identification of these proteins other bioanalytical methods are required (Q-TOF, immuno methods).

1. Sample processing—detailed elution procedures

In order to avoid the multifold process of thawing and freezing all CSF samples after centrifugation were subdivided (20μl) and transferred to 0.5 mL eppendorf vials and stored in -81°C. The Bradford method was used for the determination of total protein concentration in all analyzed samples of both patients with diagnosed subarachnoid hemorrhage and control group, respectively. The sample processing for MS analysis was strictly correlated with the data received from Bradford method. An appropriate amount of cerebrospinal fluid (CSF) was diluted with 6.0 µL of trifluoroacetic acid (TFA, 0.1%) and deionized water to obtain constant protein concentration (10 µg/mL) for all analyzed samples. The sample volume was always 20.0 µL.

The enrichment and separation procedure of proteins was performed using reversed phase ZipTip (Millipore) pipette tips (10 µL) with a bed of chromatography media (C18) fixed at its end. It was intended to use for concentrating and purifying peptides or proteins from CSF samples derived from subarachnoid haemorrhage (SAH) patents.

All CSF samples were processed by ZipTip pipette tips according to the following protocol. Equilibrating and washing of the tip end by double aspirating with 10 µL of acetonitrile (ACN) and 0.1% trifluoroacetic acid (TFA), respectively. Thereafter, the purification and sample enrichment procedure

was carried out. The 10.0 µL of CSF sample was aspirated and dispensed in the 10 cycles for maximum binding of complex mixtures on the C18 chromatography media. The ZipTip end was again washed with 10 µL of TFA (0.1%). The separated proteins/peptides were eluted from the C18 chromatographic media using elution mixture consisted of ACN and TFA (0.1%) in the volume ratio of 50:50 (ACN:TFA), v/v%. Each time, 5 µL of eluent was aspirated and dispensed in replication of 5 cycles. Application of complex elution strategy allowed for the differentiation of eluted proteins and polypeptides. Finally, the elution mixtures were mixed with the HCCA matrix in the ratio of 1:10 (eluate:HCCA), v/v% and spotted on the target plate. Each sample was spotted in replication of a tree. The ClinProt Standard (CPS) was also spotted on the target plate. The CPS was directly diluted with the matrix in 1:10 ratio (CPS:HCCA), v/v%.

2. MALDI-TOF analysis—profiling studies

The AnchorChip Standard (800 mm, Bruker, Germany) was used as a target plate. Before every spotting, the target cleaning protocol was involved. Target was sonicated for 10 min in ultrasonic bath in isopropanol and next in acetonitrile and trifluoroacetic acid mixture, respectively.

The HCCA (α-cyano-4-hydroxycinnamic acid) was used as a matrix. The stock solution of HCCA in acetone (1 mg/ml) was further diluted to 0.3 mg/mL with the ethanol and acetone mixture.

The mass spectrometry experiments were performed utilizing MALDI-TOF apparatus (UltrafleXtreme, Bruker), equipped with the FlexControl and FlexAnalysis modules, allowing for data acquisition and data/spectra treatment.

As a mass standard in the MALDI-TOF experiments, the ClinProt Standard was used. Before each MS-analysis, the apparatus was previously calibrated according to the reference masses. The reference masses included:

- angiotensin I and II (*m/z* 1297.48 Da i 1047.18 Da),
- substance-P (1348.64 Da),
- bombestin (1620.86 Da),
- ACTH clip1-17 and ACTH clip 18-39 (2094.42 Da and 2466.58 Da, respectively),
- somatostatin-28 (3149.57 Da),
- insulin (5743.56 Da),
- cytochrome C (6181.05 Da)
- ubiquitin (4283.45 Da and 8565.89 Da).

The defined masses of calibrates allowed for the proper calibration of the apparatus and covered the mass range from 1 to 10 kDa.

Ionization procedure of analyzed CSF samples was based on the proton (H^+) transfer from matrix (HCCA) to the protein/peptide molecule due to the laser excitation. Spectra were collected as a sum of excitation beams of laser radiation of a total of 2500 shots, while single laser beam was complexed from 500 light pulses with a frequency of 1000 Hz. Utilization of the ionization presets described above allowed for the acquisition of a series of MS spectra with good satisfactory intensities over the fixed mass

3. Data analysis - statistical evaluation

The ClinProTools software (version 3.0, Bruker Daltonics) was used for the evaluation of the statistical parameters of obtained of all MS spectra. All obtained spectra (in the range 1-10 kDa) were normalized and processed with the following schedule: normalization to the total ion current (TIC), recalibration using the prominent common m/z values, baseline "top hat" subtraction, minimum baseline width 10%, smoothing, signal-to-noise ratio (S/N) ≥5, peak picking and peak calculation operation. A total average spectrum was calculated from the preprocessed spectra. The spectra averaging procedure allowed for improvement of the S/N ratio during peak picking procedure as well as due the average peak list calculation, small peaks that might be omitted in a single spectrum were included to the overall profile. In all tests $p \leq 0.05$ was considered to indicate the statistical significance. The peak statistic was described by the following parameters: ; DAve – difference between the maximal and minimal average peak intensity of all classes; PTTA – p-value of t-test (2 classes) or ANOVA test (>2 classes), range 0-1, where: 0-good, 1-bad, preferable for normal distributed data; PWKW - p-value of Wilcoxon test (2 classes) or Kruskal-Wallis test (>2 classes), range 0-1, where: 0-good, 1-bad, preferable for not normal distributed data; PAD - p-value of Anderson-Darling test, gives information about normal distribution, range 0-1, where: 0-not normal distributed, 1-normal distributed data, "<"p-value less than 0.000001. Three different algorithms were used to obtain the discriminating model: genetic algorithm (GA), quick classifier (QS) and supervised neural network (SNN). Each algorithm indicated a combination of the differentiating peaks (m/z, Da). Selected sets of peptides and low molecular proteins were subjected for further experiments with the aim of their identification as a fragments of specific proteins.

4. MALDI-TOF analysis—mass identification procedure

The attempt of the identification of selected masses was made excluding the digestion protocol, and was performed according to the procedure described below.

The eluents obtained during the ZipTip procedures and containing polypeptides and low molecular proteins in the mass range 1–10 kDa were separated and fractionated directly using reversed phase nano-liquid chromatography technique (Easy nanoLC, Bruker, Germany). The nanoLC apparatus was equipped with:

- pre-column (C18, 5μm, 120Å, L=20 mm, NS-MP 10 BioSphere)
- bioanalytical nano chromatographic column C18, 75 mm × 15 cm, 3μm, 100Å (Acclaim PepMap, Thermo Scientific)

The following mobile phases were used:

- A—0.05% TFA in water
- B—0.05% TFA in 90% acetonitrile

Analytes were eluted from the analytical column at a flow rate of around 300 nL/min during a 96 minute linear gradient from 2% to 50% of mobile phase B.

The eluent was mixed with HCCA (α-cyano-4-hydroxycinnamic acid) matrix and spotted onto AnchorChip standard plates (800 mm, Bruker, Germany). The 384 fractions with a 15 second deposition were automatically collected on the target plates using the PROTEINEER (Bruker, Germany) apparatus.

The MS spectra were acquired in the mass range 700–3500 Da and analyzed using MALDI-TOF-TOF instrument (Bruker, UltrafeXtreme, Germany) using a fixed laser intensity and 2500 shots per spectrum.

As a mass standard in these experiments, the Peptide Calibration Standard II (Bruker, Germany) was used. Before each MS-analysis, the apparatus was previously calibrated according to the reference masses that included:

- bradykinin 1-7 (*m/z* 757.3992 Da),
- angiotensin I and II (1046.5418 and 1296.6848 Da, respectively),
- substance P (1347.7354 Da),
- bombesin (1619.8223 Da),
- renin substrate (1758.9326 Da),
- ACTH clip1-17 and ACTH clip 18-39 (2093.0862 and 2465.1983 Da, respectively)
- somatostatin 28 (3147.4710 Da).

The defined masses of calibrates allowed for the proper calibration of the apparatus and covered the mass range of 700–3500 Da. Based on the mass list obtained during the MS experiments, the MS-MS mode was applied. As the parent ions for the MS-MS analysis, only those masses obtained during

profiling studies were selected as statistically important markers. Because of exclusion of the digestion procedure and limitation of parent ion mass (up to 3500 Da) in the MS-MS experiments, for further identification studies only masses up to 3500 Da were taken into consideration. The identification of the proteins was performed by Mascot platform using SwissProt database, because of the well-defined human-proteome Table SM 1.

Table SM 1. The identified masses using SwissProt database.

	Accession	Protein identified SwissProt	Mass selected [Da] (mass range)	Mass Identified [Da]	Sequence	MW [Da]	Sequence cover [%]	Score
**1**	TTHY_HUMAN	Transthyretin OS=Homo sapiens GN=TTR PE=1 SV=1	**2044.52** (2038.94-2051.90)	**2041.06**<br>*1970.04*<br>*1856.94*	**A.ALLSPYSYSTTAVVTNPKE.**<br>*A.LLSPYSYSTTAVVTNPKE.-*<br>*L.LSPYSYSTTAVVTNPKE.-*	15 877	12.9	305
**2**	H1.4_HUMAN	Histone H1.4 OS=Homo sapiens GN=HIST1H1E PE=1 SV=2	**1929.51** (1925.51-1932.76)	**1945.01**	**M.SETAPAAPAAPAPAEKTPVK.K**	21 852	9.1	63.5
**3**	CO3_HUMAN	Complement C3 OS=Homo sapiens GN=C3 PE=1 SV=2	**1866.52** (1860.74-1876.10)<br>**2022.73** (2017.66-2028.75)	**1865.01**<br>**2021.13**<br>*1970.04*<br>*1605.88*<br>*1777.99*<br>*1551.81*	**R.SSKITHRIHWESASLL.R**<br>**R.SSKITHRIHWESASLLR.S**<br>*A.LLSPYSYSTTAVVTNPKE.-*<br>*I.THRIHWESASLLR.S*<br>*S.SKITHRIHWESASLL.R*<br>*R.SSKITHRIHWESA.S*	187 030	1	271
**4**	PCSK1_HUMAN	ProSAAS OS=Homo sapiens GN=PCSK1N PE=1 SV=1	**2044.52** (2038.94-2051.90)	2045.03<br>*2115.10*<br>*2072.09*<br>*1973.02*	**A.ADHDVGSELPPEGVLGALLR.V**<br>*R.AADHDVGSELPPEGVLGALLR.V*<br>*A.DHDVGSELPPEGVLGALLRV.K*<br>*A.DHDVGSELPPEGVLGALLR.V*	27 356	8.5	330
**5**	AMER3_HUMAN	APC membrane recruitment protein 3 OS=Homo sapiens GN=FAM123C PE=2 SV=2	**23452.18** (2336.23-2363.27)	**2360.20**	**Q.EGGVSASAPECRCSLLAREGLLC.G**	90 388	2.7	57
**6**	7B2_HUMAN	Neuroendocrine protein 7B2 OS=Homo sapiens GN=SCG5 PE=1 SV=2	**3516.13** (3506.27-3531.27)	**3511.71**	**R.SVNPYLQGQRLDNVVAKKSVPHFSDEDKDPE.-**	23 715	14.6	109.3
**7**	APLP1_HUMAN	Amyloid-like protein 1 OS=Homo sapiens GN=APLP1 PE=1 SV=3	**2342.18** (2336.23-2363.27)	**2344.21**<br>*1615.81*<br>*2488.26*	**R.DELAPAGTGVSREAVSGLLIMGAGG.G**<br>*R.DELAPAGTGVSREAVSG.L*<br>*R.DELAPAGTGVSREAVSGLLIMGAGGGS.L*	72 131	4.2	271.5
**8**	PLM_HUMAN	Phospholemman OS=Homo sapiens GN=FXYD1 PE=1 SV=2	**2755.26** (2744.43-2766.73)	**2764.37**	**A.ESPKEHDPFTYDYQSLQIGGLVIAG.I**	10 434	27.2	128.9
**9**	AUGN_HUMAN	Augurin OS=Homo sapiens GN=C2orf40 PE=1 SV=1	**2985.48** (2969.50-2995.09)	**2983.65**<br>*1847.82*	**R.EAPVPTKTKVAVDENKAKEFLGSLKRQ.K**<br>*R.SPYGFRHGASVNYDDY.-*	17 173	19.2	117.1
**10**	HIS3_HUMAN	Histatin-3 OS=Homo sapiens GN=HTN3 PE=1 SV=2	**1332.67** (1325.55-1339.84)	**1335.67**<br>*2624.36*<br>*1561.76*<br>*3034.51*<br>*1490.76*	**A.DSHAKRHHGYK.R**<br>*A.KRHHGYKRKFHEKHHSHRGY.R*<br>*R.KFHEKHHSHRGY.R*<br>*A.DSHAKRHHGYKRKFHEKHHSHRGY.R*<br>*A.DSHAKRHHGYKR.K*	6 145	47.1	329
**11**	SYT11_HUMAN	Synaptotagmin-11 OS=Homo sapiens GN=SYT11 PE=1 SV=2	**1915.64** (1908.02-1920.72)	**1914.02**<br>*2197.20*	**M.AEITNIRPSFDVSPVVAG.L**<br>*M.AEITNIRPSFDVSPVVAGLIG.A*	48 266	4.2	123
**12**	FGD6_HUMAN	FYVE, RhoGEF and PH domain-containing protein 6 OS=Homo sapiens GN=FGD6 PE=1 SV=2	**1104.80** (1102.18-1108.08)	**1107.52**	**R.SLDEADSENK.E**	160 714	1	51
**13**	ANGT_HUMAN	Angiotensinogen OS=Homo sapiens GN=AGT PE=1 SV=1	**1050.20** (1046.49-1058.12)	**1045.53**<br>*1296.70*	**G.DRVYIHPF.H**<br>*G.DRVYIHPFHL.V*	53 121	2.1	74.4
**14**	SCG1_HUMAN	Secretogranin-1 OS=Homo sapiens GN=CHGB PE=1 SV=2	**3201.05** (3193.48-3212.19)	**3202.32**<br>*3203.33*	**R.SSQGGSLPSEEKGHPQEESEESNVSMASLGE.K**<br>*R.SSQGGSLPSEEKGHPQEESEESNVSMASLGE.K*	78 229	4.6	74
**15**	ISK5_HUMAN	Serine protease inhibitor Kazal-type5 OS=Homo sapiens GN=SPINK5 PE=1 SV=2	**1345.46** (1339.63-1348.93)	**1347.52**<br>*1276.47*	**G.PDGKMHGNLCSM.C**<br>*A.DGKSYNNQCTM.C*	120 600	2.2	58,8

FDR (False Discovery Rate) for both identity and identity or homology threshold was set <1%;

Score is -10*Log(P), where P is the probability that the observed match is a random event. Individual ions scores > 51 indicate identity or extensive homology (p<0.05)

Peptide sequence and mass (m/z) written in bold corresponds with the mass selected by ClinProTools program, while peptide sequence and mass (m/z) written in italics corresponds with an additional polypeptides masses of identified proteins.

SUPPLEMENTARY MATERIAL (SM) 2.

Manuscript title: **CSF PROTEOMICS OF PATIENTS WITH HYDROCEPHALUS AND SUBARACHNOID HAEMORRHAGE.**

Bartosz Sokół[1], Bartosz Urbaniak[2], Bartosz Zaremba[2], Norbert Wąsik[1], Zenon J. Kokot[2], Roman Jankowski[1].

1. Department of Neurosurgery, Poznan University of Medical Sciences.
2. Department of Inorganic and Analytical Chemistry, Poznan University of Medical Sciences.

PART I.

Table SM 2. Peaks statistics - comparison of CSF samples derived from SAH patients (good outcome + poor outcome) and Control group.

Abbreviations: Mass (m/z) - mass of the selected peaks; **DAve** – difference between the maximal and minimal average peak intensity of all classes; **PTTA** – p-value of t-test (2 classes) or ANOVA test (>2 classes), range 0-1, where: 0-good, 1-bad, preferable for normal distributed data; **PWKW** - p-value of Wilcoxon test (2 classes) or Kruskal-Wallis test (>2 classes), range 0-1, where: 0-good, 1-bad, preferable for not normal distributed data; **PAD** - p-value of Anderson-Darling test, gives information about normal distribution, range 0-1, where: 0-not normal distributed, 1-normal distributed data, "<" p-value less than 0.000001 ; **SAH, Control** – average peak intensity of classes SAH and Control; **SD SAH**, **SD Control** – standard deviation of the peak intensity average of classes SAH and Control.

Mass (m/z)	DAve	PTTA	PWKW	PAD	SAH	Control	SD SAH	SD Control
4352.24	3.57	<	<	<	0.82	4.39	0.62	2.0
4470.79	2.89	<	<	<	1.05	3.94	0.77	1.7
4607.49	2.44	<	<	<	0.88	3.33	0.55	1.51
4795.5	1.98	<	<	<	0.39	2.36	0.22	1.23
4832.49	1.84	<	<	<	0.52	2.37	0.53	1.16
3966.99	5.46	<	<	<	2.01	7.47	0.81	3.53
3689.7	5.37	<	<	<	1.23	6.6	0.75	3.48
4156.12	5.92	<	<	<	1.09	7.01	0.71	3.85
4587.7	4.79	<	<	<	1.08	5.87	0.45	3.12
4809.63	8.41	<	<	<	0.74	9.15	1.33	5.66
7171.88	0.84	<	<	<	0.26	1.11	0.18	0.58
4753.71	3.73	<	<	<	0.31	4.05	0.22	2.58
6047.32	0.82	<	<	<	0.31	1.13	0.19	0.57
3807.21	3.25	<	<	<	1.72	4.96	1.14	2.29
5831.47	0.67	<	<	<	0.37	1.05	0.19	0.5
4626.08	1.36	<	<	<	0.85	2.21	0.48	0.99
5811.27	0.72	<	<	<	0.3	1.02	0.15	0.54
4740.01	2.18	<	<	<	0.31	2.49	0.19	1.65
3952.71	7.71	<	<	<	3.85	11.56	1.93	5.9
6253.56	2.35	<	<	<	0.4	2.74	0.24	1.85
5800.24	0.69	<	<	<	0.3	0.99	0.15	0.55
7055.38	1.06	<	<	<	0.26	1.32	0.17	0.88
6460.44	0.65	<	<	<	0.45	1.11	0.39	0.48
5749.76	0.66	<	<	<	0.33	0.99	0.15	0.62
7252.79	0.91	<	<	<	0.21	1.12	0.12	0.86

6292.06	0.86	<	<	<	0.28	1.14	0.13	0.81
8188.05	0.93	<	<	<	0.24	1.17	0.12	0.88
7265.66	1.14	<	<	<	0.22	1.36	0.09	1.09
6504.41	0.64	<	<	<	0.39	1.03	0.3	0.58
2524.46	2.7	<	<	0.000002	2.29	4.99	1.53	2.32
6269.89	1.17	<	<	<	0.31	1.48	0.16	1.22
3430.16	13.9	<	<	<	15.79	1.9	12.02	4.05
6437.9	0.46	<	<	<	0.43	0.88	0.3	0.43
6976	2.93	<	<	<	0.82	3.75	0.85	3.26
5866.69	1.74	<	<	<	1.07	2.81	0.82	1.92
6625.78	0.94	<	<	<	0.48	1.42	0.52	1.01
1915.69	1.04	<	<	0.000007	1.49	2.53	0.79	0.97
9740.97	0.34	<	<	<	0.12	0.46	0.06	0.41
3823.72	9.55	<	<	<	11.62	2.07	9.9	1.76
5026.54	1.39	<	<	<	2.1	0.71	1.43	0.51
3276.47	8.14	<	<	<	10.03	1.9	8.52	1.38
3476.16	84.25	<	<	<	95.86	11.61	90.91	35.39
5004.51	2.87	<	<	<	4.26	1.39	3.28	0.73
3926.35	3.01	<	<	<	4.5	7.51	2.72	3.01
2734.58	1.92	<	<	0.0245	2.51	4.42	1.88	1.7
3884.31	1.61	<	<	<	2.34	3.95	1.33	1.77
3390.36	5.75	<	<	<	7.64	1.89	6.55	2.71
2985.48	2.73	<	<	<	3.14	5.88	2.59	2.92
3371.1	7.01	<	<	<	8.48	1.48	8.76	0.71
4965.25	2.97	<	<	<	4.76	1.79	3.76	0.76
1050.35	2.39	<	<	<	1.66	4.05	1.33	3.39
5885.97	0.99	<	<	<	1.01	2	0.86	1.23
1066.57	8.32	<	<	<	2.61	10.93	3.36	12.29
3444.27	7.1	<	<	<	10.2	3.1	9.27	2.29
3857.11	5.26	<	<	<	7.21	1.95	6.99	1.66
3743.6	14.62	<	<	<	17.08	2.46	20.13	2.57
1082.43	3.77	<	<	<	1.97	5.74	1.87	5.99
1072.27	4.48	<	<	<	2.22	6.7	2.46	7.19
3497.66	14.01	<	<	<	19.41	5.4	17.94	12.9
1738.88	1.26	<	<	<	2.67	1.41	1.73	0.83
1277.63	4.09	<	0.000004	<	1.99	6.08	1.99	6.89
2675.3	2.26	<	<	<	2.26	4.52	3.07	2.02
1088.51	2.88	<	<	<	1.86	4.74	1.79	4.82
1293.61	2.54	<	<	<	1.73	4.27	1.51	4.45
3328.9	13.53	<	<	<	15.01	1.49	21.84	1.88
5065.35	0.8	<	<	<	1.62	2.42	1	1.13
1104.73	1.35	<	0.000006	<	1.07	2.41	0.96	2.48
1299.39	1.53	<	0.0000441	<	1.34	2.87	1.11	2.81
1283.21	1.99	<	0.0000012	<	1.49	3.48	1.58	3.62
3586.91	2.95	<	<	<	5.28	2.34	5.04	1.17
4987.04	1.13	<	0.0000781	<	2.68	1.55	1.94	0.67
1094.2	1.74	<	<	<	1.64	3.38	1.75	3.17
6681.23	0.88	<	<	<	0.48	1.36	0.44	1.81
1078.07	2.31	<	0.000008	<	2.08	4.4	2.15	4.54
3554.41	1.83	<	<	0.00002	3.64	5.47	2.84	2.69
2022.65	9.53	0.000004	<	<	13.69	4.16	17.95	9.77
2580.24	4.44	0.000004	0.000258	<	6.71	2.26	9.01	1.84
1866.4	1.89	0.0000662	<	<	3.23	1.34	4.38	1.41
6806.72	1.75	0.0000722	0.977	<	2.68	0.94	4.19	0.65
4938.89	0.35	0.000116	0.00528	<	1.3	0.95	0.82	0.33
1466.62	3.08	0.000134	0.000158	<	5.42	2.34	7.41	2.77
1305.43	0.91	0.000163	0.000094	<	1.53	2.44	1.45	2.15
2044.45	1.44	0.000593	0.0208	<	2.8	1.35	3.82	1.65
2939.24	4.53	0.00114	0.000962	<	9.04	4.51	12.85	4.78
1420.01	1.43	0.00115	0.622	<	2.56	1.13	4.05	1.57
5045.32	0.59	0.00217	0.000324	<	2.37	1.78	1.52	1.27
1289.25	1.1	0.00247	0.00835	<	2.02	3.12	2.35	3.06
1504.92	0.61	0.00329	0.0525	<	1.47	2.08	1.21	1.88
8571.45	0.29	0.00398	<	<	0.85	0.56	0.6	0.89
2795.22	2.43	0.00632	0.13	<	5.79	3.36	8.07	3.7
2428.95	1.65	0.00632	0.0000125	<	4.96	6.61	4.65	4.19
2342.65	0.71	0.00653	0.339	<	3.01	2.3	2.38	1.04
1332.78	0.83	0.00842	0.991	<	2.41	1.58	2.92	1.12

6844.17	0.39	0.0145	0.121	<	1.18	0.79	1.5	0.41
6884.44	0.54	0.0153	0.00326	<	0.78	1.32	0.88	2.29
1310.08	2.8	0.0184	0.409	<	5.57	2.76	11.44	2.21
1514.97	0.69	0.0285	0.295	<	2.31	1.62	2.91	1.09
5582.46	0.57	0.0288	0.000004	<	0.48	1.04	0.31	2.88
2629.22	0.82	0.0499	0.554	<	4.3	3.47	3.48	2.48
2379.88	0.63	0.0522	0.295	<	3.71	3.09	2.79	1.72
3537.11	1.28	0.0557	0.343	<	6.54	5.26	5.53	4.06
2755.23	6.11	0.0638	0.295	<	18.45	12.34	28.51	17.45
2777.07	1.08	0.125	0.0335	<	4.01	2.93	6.16	3.44
4373.97	0.79	0.129	<	<	1.35	2.14	5.01	0.78
1488.75	0.36	0.244	0.223	<	1.98	2.34	2.27	2.12
1538.01	1.23	0.249	0.000977	<	4.45	3.22	9.81	3.59
1509.41	0.3	0.263	0.879	<	2	1.7	2.25	1.43
6820.37	0.18	0.272	0.0000161	<	1.14	1.32	1.42	0.89
2329.81	0.35	0.272	0.0000141	<	2.75	3.1	2.82	1.4
3905.95	0.95	0.311	0.0584	<	9.01	9.97	7.5	5.96
7567.54	1.07	0.439	0.00118	<	3.42	2.34	8.9	11.32
1575.98	0.2	0.489	0.466	<	1.61	1.81	1.87	2.35
4567.59	0.11	0.5	0.0225	<	2.13	2.24	1.37	0.85
1554.01	0.99	0.606	0.0137	<	7.88	6.89	14.29	13.03
3515.01	1.07	0.612	0.0563	<	22.58	23.66	17.98	11.23
7938.62	0.16	0.697	0.751	<	1.09	0.92	2.47	3.64
7662.54	0.04	0.821	0.0759	<	1.1	1.14	1.25	1.16
7508.43	0.11	0.835	0.622	<	1.28	1.17	3.37	4.49
7489.21	0.08	0.92	0.16	<	1.69	1.76	5.14	7.04

Table SM 3. The discriminant masses of cerebrospinal fluid samples derived from SAH patients (without selection of Good Outcome SAH and Poor Outcome SAH individuals) and Control Group.

**Genetic Algorithm (GA)**			**Quick Classifier (QC)**			**Supervised Neural Network (SNN)**		
**Mass (m/z)**	*Start Mass*	*End mass*	**Mass (m/z)**	Start Mass	End mass	**Mass (m/z**)	Start Mass	End mass
3390.36	3383.4	3399.06	3276.47	3267.56	3291.07	5866.69	5838.34	5880.98
3743.6	3735.78	3759.14	3328.9	3321.56	3341.27	4965.25	4953.91	4975.7
2342.65	2336.79	2354.54	3371.1	3362.09	3383.4	4352.24	4340.1	4361.21
2985.48	2969.5	2995.09	3390.36	3383.4	3399.06	3430.16	3421.54	3437.08
1738.88	1735.01	1746.66	3430.16	3421.54	3437.08	3497.66	3492.46	3504.93
2580.24	2574.1	2593.29	3444.27	3437.08	3456.49	1575.98	1572.3	1580.51
3276.47	3267.56	3291.07	3476.16	3456.49	3484.31	3537.11	3530.57	3547.43
4965.25	4953.91	4975.7	3497.66	3492.46	3504.93	5582.46	5576.27	5614.93
7265.66	7258.17	7279.92	3743.6	3735.78	3759.14	4470.79	4444.45	4486.98
6047.32	6028.48	6060.47	3823.72	3814.6	3835.51	3554.41	3547.43	3569.32
3857.11	3850.38	3873.64	4352.24	4340.1	4361.21	3444.27	3437.08	3456.49
4938.89	4930.64	4945.21	5004.51	4994.71	5016.59	1072.27	1069.92	1075.5
5866.69	5838.34	5880.98	5026.54	5017.36	5034.64	3586.91	3569.32	3602.61
6806.72	6776.86	6815.91				1066.57	1062.71	1069.92
3823.72	3814.6	3835.51				3371.1	3362.09	3383.4
						1283.21	1281.11	1286.43
						6292.06	6285.63	6321.2
						3743.6	3735.78	3759.14
						2580.24	2574.1	2593.29
						4373.97	4366.49	4382.61

PART II.

Table SM 4. Peaks statistics - comparison of CSF samples derived from Good Outcome SAH (GO-SAH) patients and Control group.

Abbreviations: Mass (m/z) - mass of the selected peaks; **DAve** – difference between the maximal and minimal average peak intensity of all classes; **PTTA** – p-value of t-test (2 classes) or ANOVA test (>2 classes), range 0-1, where: 0-good, 1-bad, preferable for normal distributed data; **PWKW** - p-value of Wilcoxon test (2 classes) or Kruskal-Wallis test (>2 classes), range 0-1, where: 0-good, 1-bad, preferable for not normal distributed data; **PAD** - p-value of Anderson-Darling test, gives information about normal distribution, range 0-1, where: 0-not normal distributed, 1-normal distributed data, “<” p-value less than 0.000001 ; **GO-SAH, Control** – average peak intensity of classes GO-SAH and Control; **SD GO-SAH**, **SD Control** – standard deviation of the peak intensity average of classes GO-SAH and Control.

Mass (m/z)	DAve	PTTA	PWKW	PAD	GO-SAH	Control	SD GO-SAH	SD Control
4352.22	3.53	<	<	<	0.88	4.42	0.6	1.99
4470.76	3.01	<	<	<	0.95	3.96	0.69	1.69
4795.51	1.96	<	<	<	0.41	2.37	0.26	1.23
2675.25	3.28	<	<	<	1.27	4.55	0.72	2.01
4156.13	5.92	<	<	<	1.14	7.06	0.8	3.83
3689.71	5.39	<	<	<	1.2	6.59	0.49	3.49
4587.7	4.78	<	<	<	1.13	5.9	0.42	3.1
3966.85	5.4	<	<	<	2.1	7.5	0.79	3.53
4607.51	2.35	<	<	<	0.98	3.33	0.6	1.51
4809.64	8.38	<	<	<	0.82	9.2	1.67	5.64
6047.35	0.84	<	<	<	0.29	1.13	0.17	0.57
4753.69	3.72	<	<	<	0.35	4.07	0.21	2.58
7171.78	0.83	<	<	<	0.28	1.12	0.22	0.58
4832.5	1.85	<	<	<	0.53	2.38	0.64	1.16
3807.21	3.41	<	<	<	1.59	4.99	1.12	2.27
5811.29	0.72	<	<	<	0.3	1.03	0.13	0.54
4740.12	2.18	<	<	<	0.33	2.51	0.22	1.65
5831.4	0.67	<	<	<	0.38	1.05	0.16	0.5
6253.54	2.37	<	<	<	0.39	2.76	0.24	1.85
5799.95	0.68	<	<	<	0.31	0.99	0.13	0.55
7055.38	1.04	<	<	<	0.29	1.33	0.2	0.88
3952.54	7.26	<	<	<	4.32	11.58	2.08	5.92
4626.14	1.26	<	<	0.0000149	0.95	2.21	0.48	0.99
2524.51	3.01	<	<	0.000624	1.99	5	1.29	2.3
6292.21	0.88	<	<	<	0.26	1.14	0.13	0.81
2985.51	3.74	<	<	<	2.17	5.91	1.7	2.9
7265.53	1.14	<	<	<	0.23	1.37	0.1	1.1
7252.61	0.89	<	<	<	0.24	1.13	0.14	0.86
8187.84	0.89	<	<	<	0.26	1.15	0.14	0.87
5749.7	0.64	<	<	<	0.35	1	0.16	0.62
6504.43	0.66	<	<	<	0.37	1.04	0.28	0.58
6270.04	1.18	<	<	<	0.3	1.49	0.17	1.22
2734.5	2.53	<	<	0.0743	1.92	4.44	1.48	1.69
3234.92	2.58	<	<	0.000002	1.75	4.32	1.37	2.13
6976	3.03	<	<	<	0.73	3.77	0.66	3.26
1915.64	1.04	<	<	0.00559	1.46	2.5	0.55	0.91
9740.75	0.34	<	<	<	0.13	0.47	0.07	0.41
6625.48	0.97	<	<	<	0.48	1.45	0.57	1.01
6437.99	0.48	<	<	<	0.4	0.88	0.32	0.43
6460.38	0.63	<	<	<	0.47	1.1	0.46	0.48
3430.09	16.68	<	<	<	18.58	1.91	13.5	4.06
2329.98	1.36	<	<	0.0000915	1.75	3.11	0.94	1.4

5866.67	1.51	<	<	<	1.28	2.79	0.9	1.91
3476.1	105.84	<	<	<	117.53	11.69	97.84	35.51
3390.23	7.56	<	<	<	9.45	1.89	7.08	2.71
1066.57	8.39	<	<	<	2.42	10.81	3.12	12.25
5885.85	0.99	<	<	<	1	1.99	0.68	1.23
3276.39	10.66	<	<	<	12.53	1.87	10.36	1.36
3823.65	10.37	<	<	<	12.45	2.08	10.36	1.76
1738.89	1.65	<	<	<	3.03	1.38	1.64	0.81
3497.58	17.77	<	<	<	23.26	5.48	17.29	12.94
3884.39	1.57	<	<	0.00103	2.4	3.97	1.36	1.77
1050.32	2.22	<	0.000005	<	1.81	4.02	1.31	3.38
1082.4	3.6	<	<	<	1.99	5.59	1.85	5.84
3856.88	6.46	<	<	<	8.41	1.95	7.54	1.66
1277.63	4.03	<	<	<	2.0	6.03	2.15	6.89
1072.27	4.3	<	0.0000034	<	2.36	6.67	2.74	7.2
3586.91	4.37	<	<	<	6.59	2.22	5.65	1.18
1088.5	2.69	<	0.0000589	<	1.96	4.65	1.94	4.75
1293.54	2.37	<	0.000815	<	1.8	4.17	1.53	4.39
5025.93	1.14	<	<	<	1.84	0.71	1.5	0.5
5065.18	0.86	<	<	<	1.57	2.43	1	1.13
3743.55	16.53	<	<	<	19.02	2.5	23.03	2.57
6681.45	0.9	<	<	<	0.49	1.39	0.52	1.8
1538.05	1.77	<	<	<	1.43	3.2	1.01	3.6
3926.01	2.46	<	<	<	5.09	7.55	3.06	2.98
3444.23	7.26	0.0000014	<	<	10.37	3.11	10.65	2.29
1104.7	1.23	0.0000025	0.0000969	<	1.13	2.36	1.08	2.42
3370.75	7.85	0.0000031	0	<	9.32	1.48	12.03	0.71
5003.55	2.07	0.0000037	<	<	3.48	1.4	3.19	0.73
1299.45	1.36	0.0000052	0.00615	<	1.47	2.83	1.22	2.79
1283.24	1.81	0.0000083	0.000178	<	1.65	3.46	1.84	3.63
4964.79	2.02	0.000014	0.000041	<	3.81	1.8	3.33	0.76
6821.00	0.57	0.0000217	<	<	0.79	1.36	0.81	0.9
7825.18	0.22	0.0000626	0.0000019	<	0.63	0.41	0.36	0.32
3328.78	17.56	0.0000866	0	0	19.04	1.48	32.74	1.88
1078.07	2.05	0.000087	0.000809	<	2.37	4.42	2.5	4.55
1094.2	1.45	0.000192	0.000217	<	1.91	3.36	2.01	3.17
2698.7	1.33	0.00029	<	<	1.83	3.16	2.55	1.47
2580.29	3.65	0.00122	0.0311	<	5.92	2.27	8.29	1.85
1332.61	1.5	0.00171	0.259	<	3.13	1.64	3.48	1.13
3537.13	2.43	0.00179	0.00611	<	7.71	5.28	5.18	4.07
6884.13	0.65	0.00312	0.00248	<	0.66	1.31	0.59	2.29
4987.16	0.79	0.00365	0.253	<	2.35	1.56	1.98	0.66
3554.5	1.2	0.00415	0.000886	0.00395	4.3	5.5	2.59	2.69
2428.95	1.88	0.00535	0.000302	<	4.83	6.7	4.13	4.45
6862.05	0.24	0.00561	0.0142	<	0.64	0.88	0.43	0.75
2477.52	0.74	0.00908	<	<	2.37	3.11	1.98	1.21
2342.42	0.91	0.0131	0.272	<	3.21	2.3	2.67	1.05
6805.93	0.79	0.0131	0.132	<	1.6	0.81	2.38	0.59
2022.68	4.94	0.0132	0.000144	<	9.11	4.17	13.59	9.81
1466.53	2.67	0.0132	0.01	<	5.01	2.34	7.94	2.78
8570.78	0.26	0.0201	0.0000013	<	0.81	0.55	0.58	0.9
1420.24	1.06	0.0268	0.297	<	2.19	1.12	3.48	1.57
5582.88	0.58	0.0269	0.0000102	<	0.45	1.04	0.33	2.89
1309.79	3.8	0.0315	0.844	<	6.53	2.73	13.3	2.18
1305.35	0.6	0.0393	0.0393	<	1.83	2.43	1.68	2.16
4938.47	0.22	0.0445	0.377	<	1.17	0.95	0.78	0.33
5045.03	0.47	0.0641	0.177	<	2.25	1.79	1.71	1.27
1553.99	2.31	0.0881	0.15	<	4.61	6.91	5.03	13.08
8299.38	0.09	0.116	0.468	<	0.42	0.51	0.33	0.41
1509.13	0.57	0.143	0.932	<	2.35	1.78	2.74	1.53
2755.24	3.55	0.152	0.0000075	<	8.87	12.42	14.47	17.49
1289.15	0.61	0.191	0.463	<	2.59	3.19	2.77	3.12
3515.11	3.23	0.201	0.588	<	26.99	23.77	17.51	11.2
7567.7	1.76	0.261	<	<	4.11	2.36	8.82	11.36
2379.77	0.37	0.353	0.97	<	3.47	3.1	2.75	1.72
3905.22	1.11	0.36	0.686	<	11.12	10.02	8.06	5.95
7938.64	0.4	0.435	0.000795	<	1.33	0.93	2.85	3.66
7508.18	0.54	0.436	0.0745	<	1.73	1.19	4.15	4.5

6842.71	0.07	0.554	0.0113	<	0.86	0.78	0.87	0.39
2939.07	0.56	0.564	0.855	<	5.1	4.54	6.42	4.79
1488.78	0.22	0.598	0.377	<	2.11	2.33	2.67	2.12
7489.12	0.56	0.604	0.932	<	2.33	1.77	6.42	7.06
4567.3	0.1	0.619	0.377	0.0000322	2.35	2.26	1.39	0.85
2629.29	0.2	0.636	0.972	<	3.69	3.49	2.78	2.48
4373.49	0.36	0.662	<	<	1.79	2.15	6.33	0.78
7662.3	0.06	0.689	0.754	<	1.14	1.08	0.95	0.96
2795.1	0.05	0.93	0.0791	<	3.32	3.37	3.75	3.71

Table SM 5. The discriminant masses of cerebrospinal fluid samples derived from Good Outcome SAH patients and Control Group.

**Genetic Algorithm (GA)**			**Quick Classifier (QC)**			**Supervised Neural Network (SNN)**		
**Mass (m/z)**	*Start Mass*	*End mass*	**Mass (m/z)**	Start Mass	End mass	**Mass (m/z)**	Start Mass	End mass
4938.47	4930.48	4945.05	3276.39	3267.78	3290.86	3430.09	3421.54	3437.07
2734.5	2724.33	2744.45	3328.78	3321.57	3341.49	3497.58	3492.46	3505.14
3537.13	3530.78	3547.42	3370.75	3362.1	3383.19	3537.13	3530.78	3547.42
3276.39	3267.78	3290.86	3390.23	3383.19	3399.27	3554.5	3547.42	3569.31
7265.53	7258.18	7279.62	3430.09	3421.54	3437.07	3476.1	3458.83	3484.52
1066.57	1062.58	1069.91	3444.23	3437.07	3456.27	3390.23	3383.19	3399.27
1738.89	1735.04	1743.05	3476.1	3458.83	3484.52	3276.39	3267.78	3290.86
3390.23	3383.19	3399.27	3497.58	3492.46	3505.14	3444.23	3437.07	3456.27
7825.18	7812.98	7852.99	3743.55	3735.54	3758.89	2734.5	2724.33	2744.45
2939.07	2929.1	2947.21	3823.65	3814.58	3835.48	8299.38	8286.88	8334.41
2428.95	2418.75	2437.34	4352.22	4340.02	4361.13	5866.67	5838.32	5880.95
5866.67	5838.32	5880.95				3586.91	3578.87	3602.82
5065.18	5055.15	5081.05				5065.18	5055.15	5081.05
4587.7	4575.98	4598.64				4352.22	4340.02	4361.13
2985.51	2969.33	2993.91				2477.52	2460.15	2485.24
						6625.48	6592.35	6651.62
						1915.64	1908.02	1920.72
						4964.79	4953.75	4975.54
						3823.65	3814.58	3835.48
						5885.85	5880.95	5925.98
						7825.18	7812.98	7852.99
						6047.35	6028.43	6060.7

PART III.

Table SM 6. Peaks statistics - comparison of CSF samples derived from Poor Outcome SAH (PO-SAH) patients and Control group.

Abbreviations: Mass (m/z) - mass of the selected peaks; **DAve** – difference between the maximal and minimal average peak intensity of all classes; **PTTA** – p-value of t-test (2 classes) or ANOVA test (>2 classes), range 0-1, where: 0-good, 1-bad, preferable for normal distributed data; **PWKW** - p-value of Wilcoxon test (2 classes) or Kruskal-Wallis test (>2 classes), range 0-1, where: 0-good, 1-bad, preferable for not normal distributed data; **PAD** - p-value of Anderson-Darling test, gives information about normal distribution, range 0-1, where: 0-not normal distributed, 1-normal distributed data, “<” p-value less than 0.000001 ; **PO-SAH, Control** – average peak intensity of classes PO-SAH and Control; **SD PO-SAH**, **SD Control** – standard deviation of the peak intensity average of classes PO-SAH and Control.

Mass (m/z)	DAve	PTTA	PWKW	PAD	PO-SAH	Control	SD PO-SAH	SD Control
4352.27	3.68	<	<	<	0.68	4.36	0.6	2.02
4607.49	2.62	<	<	<	0.7	3.32	0.4	1.5
4795.45	2.03	<	<	<	0.32	2.35	0.19	1.23
4832.49	1.9	<	<	<	0.45	2.36	0.27	1.17
3967.08	5.63	<	<	<	1.81	7.44	0.81	3.54
4587.68	4.86	<	<	<	0.97	5.84	0.45	3.13
4156.14	5.95	<	<	<	1.02	6.97	0.72	3.87
7171.99	0.89	<	<	<	0.21	1.09	0.11	0.58
3689.7	5.38	<	<	<	1.23	6.61	1.16	3.45
4809.65	8.56	<	<	<	0.53	9.09	0.32	5.69
4753.73	3.78	<	<	<	0.24	4.02	0.21	2.59
3952.95	8.23	<	<	<	3.31	11.54	1.5	5.89
4470.85	2.84	<	<	0.000003	1.08	3.92	0.96	1.71
4740.15	2.2	<	<	<	0.28	2.48	0.19	1.65
6253.62	2.36	<	<	<	0.36	2.73	0.25	1.86
5811.41	0.74	<	<	<	0.28	1.02	0.2	0.54
7055.44	1.11	<	<	<	0.21	1.31	0.09	0.88
5801.31	0.72	<	<	<	0.26	0.99	0.18	0.55
5831.33	0.71	<	<	<	0.33	1.04	0.24	0.5
6047.26	0.79	<	<	<	0.34	1.13	0.3	0.57
5749.78	0.72	<	<	<	0.27	0.99	0.15	0.62
3807.19	3.03	<	<	<	1.91	4.93	1.11	2.31
7252.7	0.95	<	<	<	0.16	1.11	0.1	0.86
5866.74	2.17	<	<	<	0.63	2.8	0.5	1.92
4625.92	1.5	<	<	<	0.7	2.2	0.65	0.99
8188.09	0.97	<	<	<	0.19	1.16	0.12	0.88
6460.56	0.71	<	<	<	0.4	1.1	0.31	0.48
6292.05	0.88	<	<	<	0.26	1.13	0.14	0.81
7265.82	1.15	<	<	<	0.21	1.35	0.12	1.09
1022.56	1.05	<	<	0.00791	0.81	1.86	0.52	0.72
6269.99	1.17	<	<	<	0.29	1.47	0.17	1.22
9740.97	0.37	<	<	<	0.09	0.46	0.05	0.41
6975.95	2.77	<	<	<	0.96	3.72	1.08	3.26
1050.41	2.97	<	<	<	1.16	4.12	1.21	3.48
1034.1	1.01	<	<	<	0.87	1.88	0.52	1.15
3926.77	3.46	<	<	<	4.01	7.47	2.35	3.03
2524.47	2.54	<	<	0.017	2.45	4.99	1.75	2.31
1094.21	2.43	<	<	<	0.99	3.42	1	3.2

1072.29	5.34	<	<	<	1.54	6.89	1.73	7.43
1066.6	9.01	<	<	<	2.21	11.22	3.31	12.69
1289.62	2.11	<	<	<	0.93	3.04	0.77	2.97
1082.49	4.27	<	<	<	1.53	5.79	1.68	6.02
1088.55	3.48	<	<	<	1.35	4.84	1.39	4.91
1293.72	3.16	<	<	<	1.21	4.37	1.17	4.5
1283.17	2.58	<	<	<	1	3.58	0.88	3.71
1028.33	0.91	<	<	<	0.87	1.78	0.65	0.97
1299.36	1.97	<	<	<	0.95	2.92	0.77	2.86
1078.07	3.08	<	<	<	1.36	4.43	1.21	4.55
1305.62	1.53	<	<	<	0.93	2.46	0.78	2.17
1277.68	4.66	<	<	<	1.59	6.25	1.59	7.15
3444.24	7.67	<	<	<	10.74	3.08	7.22	2.29
3276.63	5.41	<	<	<	7.33	1.92	5.11	1.39
3430.23	13.56	<	<	<	15.45	1.89	12.93	4.03
1104.8	1.61	<	0.000001	<	0.84	2.45	0.74	2.51
5026.76	1.51	<	<	<	2.22	0.71	1.47	0.51
6504.92	0.57	<	<	<	0.46	1.03	0.5	0.57
5886.13	1.16	<	<	<	0.85	2	0.99	1.22
5004.89	3.61	<	<	<	4.99	1.38	3.66	0.73
3371.28	7.02	<	<	<	8.49	1.48	7.26	0.71
1110.28	1.09	<	0.000005	<	0.87	1.96	0.6	1.76
3823.8	9.41	<	<	<	11.47	2.06	9.92	1.75
6437.52	0.43	<	<	0.000017	0.46	0.89	0.42	0.43
4965.3	4.07	<	<	<	5.86	1.78	4.7	0.76
6073.95	0.35	<	<	0.000906	0.41	0.76	0.38	0.35
1504.72	1.03	<	<	<	1.1	2.13	0.67	1.89
3743.77	12.45	<	<	<	14.87	2.42	15.46	2.57
3476.19	76.93	<	<	<	88.46	11.53	96.23	35.27
2734.64	1.72	<	<	0.179	2.69	4.4	2.02	1.71
3329.01	11.82	<	<	<	13.31	1.49	15.26	1.87
3857.38	5.87	0.000007	0.000027	<	7.81	1.94	8.74	1.66
3390.24	6.39	0.000008	<	<	8.28	1.89	9.52	2.7
2675.37	1.97	0.000008	<	0.00135	2.53	4.5	2.78	2.03
6679.06	0.82	0.000009	<	<	0.49	1.31	0.64	1.8
2985.45	2.22	0.00001	<	0.000004	3.61	5.83	2.99	2.94
3884.16	1.28	0.00001	0.000011	0.000013	2.66	3.94	1.7	1.77
5571.88	0.53	0.000018	<	<	0.45	0.98	0.25	1.3
4986.74	1.35	0.000019	0.000417	<	2.89	1.54	2.12	0.67
5065.51	0.73	0.000041	0.000029	<	1.68	2.41	1.04	1.14
3497.79	14.42	0.000057	0.000002	<	19.8	5.38	23.54	12.86
2022.65	12.08	0.00006	<	<	16.21	4.14	19.97	9.74
6806.44	4.18	0.000186	0.319	<	5.31	1.13	7.71	0.75
1866.42	3.19	0.000216	<	<	4.52	1.33	5.92	1.41
2580.18	4.49	0.000427	0.000702	<	6.74	2.25	8.8	1.84
2939.34	8.31	0.000453	0.000008	<	12.8	4.49	16.28	4.77
1488.69	0.9	0.000559	0.00825	<	1.47	2.37	1.26	2.19
7662.84	0.5	0.000665	<	<	0.62	1.12	0.75	1.17
3554.42	1.85	0.00131	<	0.000078	3.6	5.45	3.71	2.7
6844.69	1.13	0.00236	0.557	<	1.95	0.83	2.59	0.48
4939.28	0.4	0.00243	0.0119	<	1.35	0.95	0.9	0.33
2044.41	1.99	0.00343	0.0024	<	3.35	1.36	4.71	1.64
1466.67	2.51	0.00374	0.0196	<	4.86	2.35	5.9	2.76
2428.96	2.14	0.00399	0.000015	<	4.39	6.53	4.71	3.97
2795.16	4.36	0.00411	0.00402	<	7.7	3.34	10.55	3.69
7742.25	0.16	0.00561	0.00595	<	0.42	0.57	0.28	0.46
3906.61	2.82	0.00843	0.00043	<	7.1	9.91	6.69	5.97
2755.26	12.35	0.0113	0.395	<	24.61	12.26	33.11	17.41
1351.3	0.78	0.0125	0.621	<	2.02	1.24	2.2	0.65
5045.49	0.54	0.014	0.00196	<	2.31	1.77	1.35	1.27

1515.07	1.68	0.0207	0.347	<	3.35	1.67	5.17	1.15
1419.72	1.47	0.0207	0.515	<	2.6	1.13	4.45	1.56
1617.73	1.07	0.0223	0.0521	<	2.21	1.14	3.32	0.92
5582.14	0.59	0.0223	0.000029	<	0.46	1.05	0.32	2.87
6633.04	0.42	0.0249	<	<	0.75	1.17	1.26	0.75
2777.09	2.21	0.0366	0.897	<	5.12	2.91	7.33	3.43
1537.89	3.53	0.0491	0.1	<	6.76	3.23	12.75	3.58
8572.32	0.21	0.0657	0.00402	<	0.76	0.56	0.6	0.89
7489.64	1.13	0.0741	0.116	<	0.51	1.63	0.75	7.03
7509.29	0.71	0.0795	0.0188	<	0.43	1.14	0.72	4.48
6884.31	0.39	0.122	0.0386	<	0.93	1.32	1.1	2.28
4567.75	0.32	0.13	0.00376	0.0282	1.91	2.23	1.44	0.86
3537.38	1.53	0.156	0.575	<	6.77	5.23	7.43	4.05
7938.92	0.43	0.227	<	<	0.48	0.92	1.17	3.63
2629.08	0.62	0.296	0.228	<	4.08	3.46	3.98	2.47
1553.99	2.8	0.315	0.157	<	9.66	6.86	18.46	12.99
1310.43	1.21	0.345	0.00471		3.99	2.79	9.19	2.22
7567.52	0.84	0.483	0.955		1.48	2.32	4.67	11.29
7585.5	0.49	0.535	0.000408		1.22	1.7	3.54	6.89
2380	0.25	0.535	0.614		3.32	3.07	2.75	1.73
3515.05	0.85	0.815	0.00379		22.69	23.54	24.5	11.27
2329.59	0.09	0.859	0.00102		3.19	3.1	3.58	1.39
1575.91	0.01	0.989	0.307		1.81	1.81	2.35	2.34

Table SM 7. The discriminant masses of cerebrospinal fluid samples derived from Poor Outcome SAH patients and Control Group.

**Genetic Algorithm (GA)**			**Quick Classifier (QC)**			**Supervised Neural Network (SNN)**		
**Mass (m/z)**	*Start Mass*	*End mass*	**Mass (m/z)**	Start Mass	End mass	**Mass (m/z)**	Start Mass	End mass
3857.38	3850.67	3873.71	3276.63	3267.4	3291.53	3497.79	3492.52	3504.98
2985.45	2980.24	2996.32	3329.01	3321.19	3341.11	3430.23	3421.6	3436.92
3884.16	3873.71	3891.6	3371.28	3361.93	3383.24	3554.42	3547.49	3569.38
3276.63	3267.4	3291.53	3430.23	3421.6	3436.92	3276.63	3267.4	3291.53
2380.00	2373.1	2389.39	3476.19	3459.96	3484.36	3537.38	3530.63	3547.49
3476.19	3459.96	3484.36	4352.27	4339.94	4367.29	3444.24	3436.92	3456.54
6460.56	6448.53	6484.27				2734.64	2724.34	2744.09
7265.82	7258.05	7279.8				3823.8	3814.67	3835.8
2580.18	2574.32	2592.96				4965.3	4953.5	4976.05
5866.74	5837.91	5873.02				1537.89	1533.9	1545.28
5004.89	4995.06	5017.72				5004.89	4995.06	5017.72
3926.77	3915.9	3940.05				3371.28	3361.93	3383.24
3430.23	3421.6	3436.92				3926.77	3915.9	3940.05
4986.74	4976.05	4995.06				3743.77	3736.06	3752.08
2777.09	2771.35	2785.13				1419.72	1416.03	1423.68
						1866.42	1861.89	1876.47
						1351.3	1346.67	1356.66
						6806.44	6768.34	6817.89
						1104.8	1102.18	1108.08

PART IV.

Table SM 8. Peaks statistics - comparison of CSF samples derived from Poor Outcome SAH (PO-SAH) and Good Outcome SAH (GO-SAH) patients.

Abbreviations: Mass (m/z) - mass of the selected peaks; **DAve** – difference between the maximal and minimal average peak intensity of all classes; **PTTA** – p-value of t-test (2 classes) or ANOVA test (>2 classes), range 0-1, where: 0-good, 1-bad, preferable for normal distributed data; **PWKW** - p-value of Wilcoxon test (2 classes) or Kruskal-Wallis test (>2 classes), range 0-1, where: 0-good, 1-bad, preferable for not normal distributed data; **PAD** - p-value of Anderson-Darling test, gives information about normal distribution, range 0-1, where: 0-not normal distributed, 1-normal distributed data, “<” p-value less than 0.000001 ; **PO-SAH, GO-SAH** – average peak intensity of classes PO-SAH and GO-SAH; **SD PO-SAH**, **SD GO-SAH** – standard deviation of the peak intensity average of classes PO-SAH and GO-SAH.

Mass (m/z)	DAve	PTTA	PWKW	PAD	PO-SAH	GO-SAH	SD PO-SAH	SD GO-SAH
2032.93	2.74	0.0000224	0.0000662	<	3.98	1.24	3.7	1.21
5867.21	0.62	0.0000855	0.0000662	<	0.61	1.23	0.49	0.89
1332.65	1.96	0.00203	0.00452	<	1.29	3.24	1.34	3.63
8301.22	0.19	0.00217	0.00139	<	0.25	0.44	0.17	0.36
7745.64	0.24	0.00269	0.00289	<	0.41	0.65	0.3	0.4
3587.2	2.91	0.00271	0.00283	<	3.37	6.28	2.65	5.58
2939.1	6.95	0.00886	0.00396	<	11.82	4.86	14.58	6.25
2755.28	14.3	0.0116	0.00727	<	22.68	8.38	31.79	14.06
3276.71	4.85	0.0116	0.0507	<	7.43	12.28	5.23	10.62
5194.96	0.46	0.0116	0.0103	<	0.92	0.46	1.06	0.29
7827.99	0.2	0.0116	0.000902	<	0.39	0.59	0.35	0.34
6806.19	3.06	0.0212	0.232	<	4.78	1.72	7.44	2.79
3903.72	3.86	0.0216	0.00918	<	6.99	10.85	6.7	8.01
1537.9	4.5	0.0259	0.141	<	6.13	1.63	12.17	2.21
2795.14	3.94	0.0259	0.00283	<	7.23	3.29	10.07	3.68
2777.13	2.76	0.0259	0.0315	<	4.83	2.07	7	2.89
1866.52	2.18	0.0259	0.0887	<	4.18	2	5.68	1.86
1078.03	0.94	0.0259	0.0507	<	1.33	2.27	1.19	2.44
3944.01	0.89	0.0259	0.0671	0.0000742	3.27	4.17	1.49	2.1
4587.96	0.21	0.0314	0.00861	0.000543	0.91	1.12	0.46	0.43
4965.18	1.88	0.0369	0.0825	<	5.52	3.64	4.61	3.29
7507.19	1.28	0.0425	0.000902	<	0.39	1.67	0.62	3.97
7953.41	0.7	0.0425	<	<	0.47	1.17	0.93	1.99
5005.15	1.52	0.0476	0.0825	<	4.89	3.37	3.72	3.11
7565.94	3.21	0.055	0.000348	<	1.33	4.54	4.42	9.68
7488.28	1.79	0.0653	0.00727	<	0.48	2.27	0.59	6.16
7937.18	0.87	0.0653	<	<	0.44	1.31	1.11	2.8
6845.89	0.81	0.0653	0.896	<	1.78	0.96	2.5	1.07
1738.97	0.7	0.0766	0.00727	<	2.24	2.94	1.85	1.64
3317.18	1.37	0.0881	0.14	<	2.49	3.86	1.68	4.76
1088.5	0.63	0.0894	0.0671	<	1.28	1.9	1.34	1.9
1515.19	1.4	0.0945	0.516	<	3.11	1.71	4.93	1.39
2022.72	6.2	0.0951	0.0923	<	14.91	8.71	19.23	13.21
2044.53	1.32	0.0951	0.243	<	3.15	1.83	4.49	1.96
1072.35	0.79	0.117	0.108	<	1.45	2.24	1.67	2.68
3926.16	0.95	0.124	0.163	<	3.98	4.93	2.4	3.03
4567.8	0.5	0.128	0.0206	<	1.86	2.36	1.51	1.38
1488.89	0.75	0.134	0.243	<	1.49	2.24	1.3	2.93
3608.75	0.68	0.134	0.279	<	2.54	3.21	1.63	2.39
3966.12	0.27	0.134	0.141	0.236	1.78	2.06	0.82	0.79
8925.78	0.11	0.134	0.896	<	0.29	0.17	0.46	0.12
6461.95	0.11	0.134	0.397	<	0.31	0.42	0.21	0.43

2628.83	1.8	0.148	0.998	<	5.35	3.56	7.28	2.73
1082.37	0.55	0.148	0.0336	<	1.4	1.95	1.61	1.84
1553.94	4.15	0.159	0.896	<	8.76	4.61	17.62	5.16
6519.12	0.19	0.196	0.754	<	0.65	0.45	0.76	0.51
1310.06	3.24	0.224	0.0229	<	3.68	6.92	8.72	14.05
4986.79	0.58	0.224	0.314	<	2.83	2.26	2.15	1.95
1277.37	0.51	0.226	0.1	<	1.48	1.99	1.53	2.13
3476.36	26.91	0.23	0.0507	<	89.73	116.64	99.42	97.34
2342.69	0.65	0.234	0.14	<	2.49	3.13	2.16	2.65
3879.63	0.43	0.234	0.593	<	2.79	2.36	1.79	1.32
5026.72	0.41	0.234	0.243	<	2.26	1.84	1.61	1.49
6634.06	0.24	0.275	0.612	<	0.7	0.47	1.2	0.55
5157.09	0.2	0.277	0.896	<	0.76	0.96	0.55	1.04
6255.5	0.06	0.28	0.314	<	0.32	0.37	0.21	0.24
4939.14	0.2	0.288	0.304	<	1.32	1.12	0.88	0.78
3556.79	0.74	0.311	0.0192	<	3.47	4.2	3.63	2.57
4372.96	1	0.311	0.998	<	0.7	1.7	1.29	6.11
3765.45	0.88	0.311	0.0192	<	4.37	3.49	3.5	4.2
3430.35	2.95	0.32	0.293	<	15.6	18.55	13	13.33
4419.61	0.37	0.335	0.0671	<	1.6	1.23	1.63	1.82
5887.58	0.18	0.335	0.0825	<	0.79	0.97	0.95	0.67
3329.07	5.64	0.39	0.293	<	14.86	20.49	18.59	35.87
3350.54	1.65	0.39	0.896	<	4.16	5.82	4.04	11.29
8574.63	0.1	0.39	0.314	<	0.62	0.72	0.51	0.52
3516.49	4.03	0.408	0.0407	<	22.48	26.51	24.34	17.3
5554.85	0.04	0.42	0.341	<	0.41	0.46	0.24	0.25
3743.81	3.35	0.458	0.519	<	14.59	17.94	15.85	22.53
3497.89	3.41	0.472	0.0554	<	19.48	22.89	22.87	16.99
6884.61	0.14	0.492	0.538	<	0.81	0.67	1.05	0.62
3538.47	1	0.494	0.0507	<	6.61	7.61	7.26	5.1
6657.77	0.09	0.494	0.756	<	0.48	0.4	0.66	0.38
3411.78	0.35	0.561	0.932	<	3.04	3.39	1.99	2.9
3390.34	1.16	0.569	0.0791	<	8.56	9.72	9.37	7.42
5537.76	0.07	0.569	0.351	<	0.49	0.56	0.49	0.53
6960.66	0.11	0.605	0.651	<	0.88	0.77	1.05	0.74
6719.64	0.04	0.605	0.14	<	0.49	0.54	0.39	0.29
6674.78	0.07	0.687	0.896	<	0.55	0.48	0.83	0.5
3371.12	1.06	0.693	0.896	<	8.63	9.68	7.09	12.95
1066.58	0.3	0.697	0.151	<	2.02	2.32	3.17	3.03
6435.58	0.04	0.697	0.998	<	0.42	0.39	0.41	0.32
3857.18	0.63	0.797	0.314	<	7.91	8.55	9	7.7
3443.68	0.64	0.802	0.207	<	10.21	9.57	6.94	10.35
2580.39	0.55	0.816	0.762	<	6.34	5.79	8.4	8.03
5107.39	0.04	0.816	0.998	<	0.83	0.79	0.63	0.62
5579.18	0.02	0.816	0.834	<	0.47	0.44	0.34	0.35
1466.62	0.33	0.871	0.998	<	4.49	4.82	5.67	7.73
1419.9	0.19	0.871	0.243	<	2.41	2.22	4.24	3.66
5938.52	0.02	0.871	1	<	0.47	0.45	0.37	0.31
6476.85	0.01	0.884	0.892	<	0.33	0.35	0.28	0.29
3823.88	0.32	0.933	0.704	<	11.44	11.75	10.2	10.31
3845.69	0.06	0.954	0.883	<	3.23	3.17	2.37	2.18
3783.67	0.06	0.967	0.109	<	3.56	3.49	3.43	4.13
2380.31	0.05	0.967	0.896	<	3.28	3.34	2.71	2.71
1351.35	0.03	0.967	0.609	<	1.87	1.9	2.12	1.91
5044.7	0.02	0.967	0.704	<	2.29	2.32	1.43	1.82
5066.79	0.01	0.978	0.927	0.0000027	1.49	1.49	0.94	0.94
2424.45	0.03	0.98	0.754	<	4.41	4.44	5.96	5.19
7775.7	0	0.98	0.0123	<	0.44	0.44	0.7	0.26

Table SM 9. The discriminant masses of cerebrospinal fluid samples derived from Poor Outcome SAH and Good Outcome SAH (GO-SAH) patients.

Genetic Algorithm (GA)			Quick Classifier (QC)			Supervised Neural Network (SNN)		
**Mass (m/z)**	*Start Mass*	*End mass*	**Mass (m/z)**	Start Mass	End mass	**Mass (m/z)**	Start Mass	End mass
8301.22	8287.7	8371.9	1332.65	1325.55	1339.84	5867.21	5822.06	5880.54
7745.64	7720.86	7763.2	1738.97	1734.99	1742.7	3276.71	3267.32	3291.24
1078.03	1075.11	1080.46	2032.93	2029.29	2039.27	3926.16	3916.69	3935.37
3276.71	3267.32	3291.24	2755.28	2743.64	2765.55	5554.85	5546.81	5567.7
1738.97	1734.99	1742.7	2795.14	2784.87	2808.29	1488.89	1484.57	1492.54
5157.09	5146.94	5170.98	2939.1	2935.14	2946.57	6634.06	6595.24	6646.51
3765.45	3759.09	3774.71	3587.2	3570.37	3597.56	4567.8	4558.07	4577.98
5938.52	5928.93	5969.93	5867.21	5822.06	5880.54	1537.9	1533.71	1545.09
3879.63	3873.82	3889.44	7507.19	7501.77	7514.08	2580.39	2573.89	2592.89
5554.85	5546.81	5567.7	7565.94	7559.33	7571.7	3556.79	3549.12	3570.37
3411.78	3406.22	3418.32	7745.64	7720.86	7763.2	3966.12	3959.34	3977.2
4939.14	4917.52	4945.62	7827.99	7798.57	7829.83	2032.93	2029.29	2039.27
4567.8	4558.07	4577.98	7937.18	7916.2	7942.82	1866.52	1860.74	1876.1
3443.68	3437.25	3449.83	7953.41	7950.3	8085.13	7745.64	7720.86	7763.2
3944.01	3935.37	3959.34	8301.22	8287.7	8371.9	3823.88	3814.78	3834.56
						6719.64	6703.95	6734.41
						1553.94	1549.79	1561.37
						2755.28	2743.64	2765.55
						5107.39	5100.84	5123.99
						5537.76	5527.31	5546.81
						7775.7	7763.2	7798.57
						3443.68	3437.25	3449.83
						8301.22	8287.7	8371.9
						3538.47	3531.39	3549.12

SUPPLEMENTARY MATERIAL (SM) 3.

Manuscript title: **CSF PROTEOMICS OF PATIENTS WITH HYDROCEPHALUS AND SUBARACHNOID HAEMORRHAGE.**

Bartosz Sokół[1], Bartosz Urbaniak[2], Bartosz Zaremba[2], Norbert Wąsik[1], Zenon J. Kokot[2], Roman Jankowski[1].

1. Department of Neurosurgery, Poznan University of Medical Sciences.
2. Department of Inorganic and Analytical Chemistry, Poznan University of Medical Sciences.

PART 1.

Table SM 10. Peak statistics for samples derived from Good Outcome SAH (GO-SAH) patients. Comparison of CSF samples collected at days 1, 5 and 10 after the SAH incident.

Abbreviations: Mass (m/z) - mass of the selected peaks; **DAve** – difference between the maximal and minimal average peak intensity of all classes; **PTTA** – p-value of t-test (2 classes) or ANOVA test (>2 classes), range 0-1, where: 0-good, 1-bad, preferable for normal distributed data; **PWKW** - p-value of Wilcoxon test (2 classes) or Kruskal-Wallis test (>2 classes), range 0-1, where: 0-good, 1-bad, preferable for not normal distributed data; **PAD** - p-value of Anderson-Darling test, gives information about normal distribution, range 0-1, where: 0-not normal distributed, 1-normal distributed data, “<”p-value less than 0.000001 ; **GO-Day** – peak intensity average of classes GO-SAH Day1, Day5 and Day10; **SD GO-Day** – standard deviation of the peak intensity average of classes GO-SAH Day1, Day5 and Day10.

Mass (m/z)	DAve	PTTA	PWKW	PAD	GO-Day1	GO-Day5	GO-Day10	SD GO-Day1	SD GO-Day5	SD GO-Day10
3497.53	24.03	0.000238	0.000177	1.13E-06	11.3	24.3	35.33	10.18	13.88	21.81
3538.04	6.84	0.000238	0.000555	2.86E-06	4.15	8.41	11	2.62	4.69	6.41
3556.26	3.35	0.000238	0.00112	1.28E-05	2.5	4.48	5.85	1.1	2.44	3.05
1929.51	1.16	0.000238	0.0016	<	1.21	2.18	2.37	0.47	1.83	0.96
3516.13	22.46	0.00139	0.0016	8.69E-05	15.26	28.42	37.72	12.75	15.71	20.91
2755.19	18.01	0.00139	0.000405	<	19.9	3.03	1.89	19.41	2.26	1.33
2938.56	7.84	0.00181	0.000177	<	9.92	2.83	2.08	8.69	1.69	0.75
2580.37	6.15	0.00181	0.000909	<	8.49	6.03	2.33	5.81	10.89	2.85
3390.12	7.06	0.00223	0.000845	<	5.33	12.12	12.39	4.56	8.65	8.2
6804.72	4.39	0.00242	0.000196	<	4.71	1.29	0.32	5.32	2.1	0.14
6823.14	1.71	0.00242	0.000405	<	2.03	0.62	0.32	2.13	0.6	0.15
2044.52	1.55	0.00242	0.0179	<	2.4	2.14	0.85	1.83	2.45	0.55
6844.63	1.74	0.00298	0.00195	<	2.17	0.72	0.43	2.09	0.65	0.25
3700.18	1.09	0.00298	0.00562	0.0152	1.47	1.84	2.56	0.82	1.25	0.93
2022.65	8.97	0.00518	0.0237	<	10.71	11.72	2.75	10.93	17.7	2.6
2794.99	4.48	0.00518	0.00173	<	6.16	1.94	1.68	5.01	0.77	1.46
3370.56	10.93	0.0132	0.0325	<	6.85	15.48	4.54	3.53	19.29	2.36
3476.08	91.83	0.0132	0.00173	<	65.57	130.17	157.4	83.21	101.68	94.51
3586.86	4.29	0.0132	0.0144	<	3.58	6.68	7.87	2.76	6.45	5.63
5536.9	0.54	0.0132	0.0144	<	0.88	0.43	0.34	0.68	0.46	0.18
6763.62	0.59	0.0134	0.00874	<	0.9	0.38	0.32	0.78	0.22	0.12
6517.9	0.68	0.0141	0.0143	<	0.93	0.35	0.25	0.92	0.36	0.17
3451.37	3.18	0.0175	0.054	<	5	8.18	6.99	2.73	3.77	4.1

2884.47	5.45	0.0283	0.00827	<	6.18	1.41	0.73	9.71	1.64	0.34
7827.12	0.31	0.0283	0.0208	0.000299	0.55	0.51	0.82	0.34	0.26	0.38
1309.76	8.24	0.0336	0.00472	<	8.28	9.62	1.38	14.84	17.23	0.73
1420.06	3.39	0.0336	0.176	<	4.37	0.98	2.07	5.26	1.13	3.4
6460.72	0.26	0.034	0.151	<	0.45	0.27	0.53	0.28	0.15	0.65
6958.38	0.95	0.0346	0.0145	<	1.4	0.55	0.45	1.43	0.45	0.27
3411.6	2.11	0.0353	0.0842	<	2.4	4.51	3.31	0.8	4.14	1.92
2342.18	1.8	0.0353	0.265	<	2.05	3.25	3.85	0.87	2.62	3.35
1088.37	1.39	0.0384	0.0742	<	2.48	1.93	1.09	2.36	1.89	0.88
6656.2	0.4	0.0402	0.0313	<	0.67	0.27	0.31	0.61	0.13	0.17
3350.25	7.06	0.0485	0.00827	<	4.35	9.48	2.41	3.84	17.3	1.5
1261.22	3.17	0.0485	0.0237	<	4.26	1.09	1.4	5.24	0.74	1.19
4372.41	3.64	0.0578	0.126	<	0.55	0.4	4.03	0.22	0.25	10.15
1866.6	1.85	0.0578	0.00544	<	3.2	1.91	1.34	2.7	1.8	1.54
6719.11	0.25	0.0578	0.0775	6.5E-06	0.68	0.43	0.53	0.42	0.19	0.21
4418.6	1.01	0.0591	0.0859	<	1.76	0.74	1.42	1.65	0.73	2.55
3276.33	8.72	0.0593	0.136	<	8.39	17.11	9.88	6.76	13.94	6.05
1094.13	1.4	0.0658	0.117	<	2.5	1.8	1.1	2.36	2.02	1.06
6254.92	0.17	0.0707	0.0325	<	0.47	0.32	0.3	0.24	0.22	0.22
6884.03	0.53	0.0722	0.0375	<	1.06	0.53	0.55	0.84	0.51	0.58
2424.22	6.55	0.0839	0.0144	<	9.57	3.82	3.02	11.8	5	2.88
1072.26	1.94	0.0839	0.0599	<	3.29	1.87	1.35	3.43	2.48	1.17
1077.98	1.75	0.0839	0.0887	<	3.21	1.99	1.47	3.02	2.33	1.16
4250.83	1.22	0.0839	0.442	<	0.87	0.8	2.01	0.49	0.51	2.16
6935.97	0.94	0.0839	0.0462	<	1.41	0.57	0.47	1.7	0.56	0.32
6632.34	0.45	0.0839	0.0998	<	0.75	0.3	0.37	0.87	0.13	0.26
6674.13	0.41	0.0839	0.062	<	0.73	0.32	0.39	0.76	0.18	0.3
8571.09	0.43	0.0938	0.0663	0.00036	0.69	0.56	0.99	0.35	0.47	0.69
3845.35	1.46	0.0973	0.0313	<	2.26	3.32	3.72	2.22	2.39	1.68
1066.46	2.03	0.105	0.0465	<	3.36	2.04	1.32	4.18	2.53	1.12
3823.5	6.38	0.108	0.0161	<	7.63	13.09	14.01	10.23	11.55	7.64
3856.72	5.52	0.12	0.00472	<	5.96	8.64	11.48	8.13	7.26	7.51
1277.18	1.56	0.12	0.0313	<	3	1.61	1.44	2.96	1.61	0.97
1466.45	5.91	0.131	0.684	<	8.68	2.77	3.16	11.94	2.03	3.58
5107.34	0.37	0.153	0.244	<	0.98	0.6	0.81	0.84	0.41	0.45
2912.18	1.29	0.158	0.405	<	3.93	2.79	2.64	2.65	1.47	0.74
4567.19	0.81	0.164	0.0599	2.33E-06	1.88	2.42	2.69	1.28	1.53	1.23
3443.41	6.64	0.164	0.2	<	6.6	13.24	7.27	2.59	15.88	2.44
5936.96	0.18	0.164	0.107	5.21E-05	0.56	0.38	0.41	0.29	0.3	0.29
3316.85	3.12	0.165	0.119	<	2.5	5.62	2.84	1.56	7.15	2.08
3903.34	4.14	0.17	0.129	2.22E-06	8.08	12.06	12.23	7.7	9.57	5.84
3297.18	1.92	0.17	0.405	<	2.23	4.15	2.42	1.37	4.28	1.19
3965.59	0.43	0.171	0.15	0.481	2.31	2.03	1.88	0.59	0.84	0.83
1082.14	1.04	0.177	0.0917	<	2.4	1.85	1.36	2.02	1.9	1.3
3328.69	22.14	0.186	0.0735	<	12.73	33.34	11.2	13.34	54.4	5.78
3878.94	0.77	0.198	0.18	0.00025	2.15	2.17	2.91	1.24	1.1	1.57
5886.54	0.34	0.2	0.0859	9.19E-06	1.19	0.88	0.85	0.64	0.78	0.57
3430.05	8.15	0.21	0.0237	<	14.48	22.63	18.43	15.45	13.17	11.16
1049.89	0.69	0.217	0.102	1.15E-06	2.16	1.55	1.48	1.35	1.37	1.14
7788.09	0.14	0.253	0.35	0.000135	0.4	0.34	0.48	0.22	0.2	0.32
5065.53	0.57	0.256	0.468	<	1.85	1.28	1.54	1.22	0.9	0.77
8300.01	0.15	0.256	0.0185	<	0.42	0.36	0.51	0.44	0.32	0.27
1509.19	0.98	0.258	0.498	<	2.63	1.65	2.6	2.55	1.54	3.45
3925.61	1.35	0.271	0.336	0.00025	4.12	5.17	5.47	2.51	3.62	2.79
4818.13	0.91	0.308	0.00472	<	1.33	0.48	0.42	2.66	0.42	0.27
1488.77	1.44	0.321	0.684	<	3.07	1.64	2.04	4.04	1.26	2.6
7488	3.18	0.348	0.244	<	1	1.48	4.18	1.35	3.28	9.8
4587.73	0.18	0.354	0.292	0.0163	1.22	1.09	1.03	0.46	0.48	0.27
7506.79	2.09	0.354	0.0609	<	0.91	1.17	3	1.52	2.56	6.1
2477.86	0.95	0.354	0.755	<	1.8	1.95	2.75	0.59	1.65	2.8
3743.38	7.8	0.358	0.525	<	17.05	21.54	13.75	28.5	23.14	9.73
5156.26	0.34	0.365	0.0242	<	1.01	0.75	1.09	1.37	0.74	0.81
3764.95	1.37	0.38	0.949	<	3.54	4.05	2.68	5.19	4.37	1.95
3608.6	0.9	0.38	0.499	<	2.68	3.15	3.57	1.31	2.71	2.67
5004.1	1.53	0.396	0.595	<	4.24	2.99	2.71	4.41	2.08	2.15
1289.01	0.91	0.398	0.439	<	2.85	1.94	2.56	2.63	1.72	3.5
4964.3	1.56	0.414	0.749	<	4.57	3.33	3.01	4.5	2.35	2.52
3783.28	1.24	0.464	0.887	<	3.48	3.96	2.72	5.2	3.92	2.54

7937.1	1.1	0.472	0.0585	<	0.78	1.1	1.88	1.33	2.28	3.95
4986.68	0.75	0.472	0.657	<	2.68	1.93	2.15	2.45	1.34	1.9
5025.74	0.6	0.472	0.684	<	2.2	1.61	1.6	2.05	0.95	1.16
7730.92	0.13	0.472	0.666	0.00224	0.53	0.49	0.61	0.28	0.27	0.39
1738.92	0.56	0.481	0.558	0.00925	2.78	3.04	3.34	1.31	1.92	1.61
4607.46	0.23	0.527	0.618	<	1.11	0.88	0.89	0.86	0.42	0.3
5043.46	0.65	0.54	0.893	<	2.67	2.02	2.11	2.28	1.58	1.42
1332.44	1.16	0.633	0.499	<	3.74	2.97	2.57	4.22	3.03	3.47
2379.84	0.6	0.672	0.887	<	3.52	2.92	3.41	2.72	2.02	3.26
5866.64	0.23	0.672	0.27	<	1.3	1.07	1.25	0.75	0.96	0.92
4937.95	0.19	0.746	0.893	1.27E-05	1.22	1.03	1.05	0.96	0.67	0.71
1553.87	1.16	0.81	0.684	<	5.04	4.33	3.88	6.61	3.86	4.54
7565.05	1.42	0.847	0.0151	<	4.03	3.37	4.8	9.97	8.15	8.23
8283.94	0.07	0.847	0.0241	<	0.37	0.3	0.31	0.49	0.31	0.11
2629.25	0.13	0.989	0.684	<	3.47	3.34	3.34	2.29	2.91	3.01
3943.77	0.03	0.999	0.893	0.0198	4.11	4.1	4.13	1.79	2.26	2.23

Table SM 11. The discriminant masses established for the Good Outcome SAH patients for the samples collected at days 1, 5 and 10 after the SAH incident.

Genetic Algorithm (GA)			Quick Classifier (QC)			Supervised Neural Network (SNN)		
**Mass (m/z)**	*Start Mass*	*End mass*	**Mass (m/z)**	Start Mass	End mass	Mass (m/z)	Start Mass	End mass
4250.83	*4241.42*	*4260.62*	1929.51	*1925.11*	*1932.76*	3370.56	*3361.74*	*3382.41*
5107.34	*5099.88*	*5123.55*	2022.65	*2017.87*	*2029.78*	3328.69	*3321.43*	*3341.55*
3497.53	*3492.52*	*3506.06*	2044.52	*2038.94*	*2051.9*	2342.18	*2336.23*	*2353.27*
3783.28	*3774.1*	*3797.12*	2580.37	*2573.68*	*2593.05*	3443.41	*3437.14*	*3449.07*
7788.09	*7779.2*	*7797.21*	2755.19	*2743.98*	*2765.32*	3411.6	*3405.27*	*3418.42*
2912.18	*2903.9*	*2920.16*	2794.99	*2784.44*	*2807.48*	3743.38	*3728.03*	*3758.71*
3516.13	*3506.27*	*3531.27*	2938.56	*2927.82*	*2946.12*	5107.34	*5099.88*	*5123.55*
8283.94	*8262.91*	*8288.78*	3370.56	*3361.74*	*3382.41*	3497.53	*3492.52*	*3506.06*
2342.18	*2336.23*	*2363.27*	3390.12	*3382.41*	*3400.18*	3538.04	*3531.27*	*3548.56*
4567.19	*4557.51*	*4577.91*	3476.08	*3464.02*	*3484.58*	3783.28	*3774.1*	*3797.12*
			3497.53	*3492.52*	*3506.06*	3316.85	*3305.12*	*3321.01*
			3516.13	*3506.27*	*3531.27*	4250.83	*4241.42*	*4260.62*
			3538.04	*3531.27*	*3548.56*	8300.01	*8288.78*	*8369.29*
			3556.26	*3548.56*	*3570.02*	3350.25	*3341.55*	*3361.53*
			3586.86	*3570.02*	*3597.2*	3764.95	*3758.71*	*3774.1*
			3700.18	*3693.06*	*3706.98*	2044.52	*2038.94*	*2051.9*
			5536.9	*5521.35*	*5545.17*	3556.26	*3548.56*	*3570.02*
			6517.9	*6504.76*	*6530.04*	3297.18	*3290.73*	*3305.12*
			6763.62	*6744.23*	*6767.58*	2022.65	*2017.87*	*2029.78*
			6804.72	*6775.67*	*6816.8*	7730.92	*7721.14*	*7737.8*
			6823.14	*6817.1*	*6834.56*	3586.86	*3570.02*	*3597.2*
			6844.63	*6834.56*	*6858.66*	6935.97	*6912.46*	*6944.9*
						1929.51	*1925.11*	*1932.76*
						5025.74	*5015.04*	*5034.12*
						2580.37	*2573.68*	*2593.05*

PART 2.

Table SM 12. Peak statistics for samples derived from Poor Outcome SAH (PO-SAH) patients. Comparison of CSF samples collected at days 1, 5 and 10 after the SAH incident.

Abbreviations: Mass (m/z) - mass of the selected peaks; **DAve** – difference between the maximal and minimal average peak intensity of all classes; **PTTA** – p-value of t-test (2 classes) or ANOVA test (>2 classes), range 0-1, where: 0-good, 1-bad, preferable for normal distributed data; **PWKW** - p-value of Wilcoxon test (2 classes) or Kruskal-Wallis test (>2 classes), range 0-1, where: 0-good, 1-bad, preferable for not normal distributed data; **PAD** - p-value of Anderson-Darling test, gives information about normal distribution, range 0-1, where: 0-not normal distributed, 1-normal distributed data, “<” p-value less than 0.000001 ; **PO-Day** – average peak intensity of classes PO-SAH Day1, Day5 and Day10; **SD PO-Day** – standard deviation of the peak intensity average of classes PO-SAH Day1, Day5 and Day10.

Mass (m/z)	DAve	PTTA	PWKW	PAD	PO-Day1	PO-Day5	PO-Day10	SD PO-Day1	SD PO-Day5	SD PO-Day10
2044.66	3.17	1.53E-05	8.45E-06	<	3.73	3.69	0.55	2.5	7.04	0.44
3765.82	4.05	3.51E-05	0.00363	<	4.67	5.62	1.57	3.31	4.02	0.81
3350.84	3.94	3.51E-05	0.000804	<	5.5	3.56	1.57	5.13	1.56	0.72
6846.72	2.78	3.51E-05	0.000423	<	3.01	0.7	0.23	3.02	0.56	0.08
6520.37	0.91	3.51E-05	0.000171	<	1.04	0.31	0.13	0.89	0.19	0.12
3476.86	188.03	4.99E-05	1.97E-05	<	29.12	111.15	217.15	27.3	73.78	125.86
3517.15	48.31	4.99E-05	3.54E-05	<	8.31	25.14	56.62	6.3	14.68	32.57
2022.82	16.77	4.99E-05	0.000255	<	19.81	13.71	3.04	15.59	25.78	3.74
2580.47	9.11	4.99E-05	8.45E-06	<	10.09	3.32	0.98	10.2	3.15	0.65
3371.57	6.77	4.99E-05	0.0261	<	10.32	8.75	3.54	8.32	5.45	0.95
1866.52	5.81	4.99E-05	0.000423	<	6.56	2.46	0.75	7.15	1.73	0.66
3587.39	4.31	4.99E-05	1.91E-05	<	1.67	4.5	5.99	1.08	2.42	2.92
6807.14	8.17	0.000052	0.000255	<	8.45	1.43	0.28	8.95	1.79	0.19
3498.42	41.11	8.41E-05	2.27E-05	<	7.22	22.09	48.33	6.02	11.8	36.18
4054.12	3.42	8.41E-05	5.74E-05	<	3.94	0.94	0.52	3.57	0.72	0.44
3539.15	13.4	9.69E-05	1.36E-05	<	2.67	7.34	16.07	2.06	3.92	11.05
3904.37	11.54	0.000114	4.28E-05	<	3.04	8.87	14.58	2.3	6.06	7.98
2755.51	33.57	0.000141	0.000772	<	36.35	16.38	2.78	40.58	21.07	3.57
2939.41	16.15	0.000141	0.000328	<	18.65	8.22	2.5	18.8	9.76	1.77
6435.94	0.43	0.000141	0.00305	<	0.58	0.35	0.16	0.52	0.19	0.11
3784.21	3.8	0.000155	0.0106	<	3.49	5.34	1.55	4.05	3.61	0.46
1739.04	3.17	0.000203	3.15E-05	<	1.36	2.67	4.53	0.67	1.31	2.5
4568.69	2.19	0.000254	6.82E-05	<	1.04	2.33	3.22	0.87	1.57	1.47
2777.32	6.58	0.000276	0.000171	<	7.18	4.25	0.6	8.36	6.32	0.58
3557.91	6.45	0.000276	1.36E-05	<	1.59	3.91	8.05	1.96	1.93	5.4
3857.91	13.49	0.000546	0.00018	<	3.02	10.76	16.51	2.52	9.09	11.56
2795.4	10.21	0.000546	0.00107	<	12.01	4.91	1.8	14.67	4.87	0.77
6406.92	0.44	0.000546	0.00129	<	0.55	0.3	0.11	0.71	0.21	0.09
2424.5	6.15	0.000565	5.18E-05	<	7.31	1.94	1.16	7.27	1.04	1.09
5580.15	0.36	0.000597	0.00494	1.89E-05	0.57	0.44	0.21	0.4	0.19	0.16
1088.6	1.28	0.000722	0.0106	<	1.25	1.74	0.47	1.09	1.78	0.31
3846.13	2.72	0.000821	7.46E-05	0.000061	2.04	4.76	3.78	2.09	2.27	1.39
2032.55	3.94	0.000852	0.00123	1.72E-06	5.39	3.53	1.45	3.82	3.52	1.89
5145.08	0.45	0.000969	0.0282	<	0.77	0.45	0.32	0.53	0.21	0.15
3430.81	16.07	0.00133	0.00162	0.000013	9.04	20.77	25.11	6.1	13.76	15.82
3390.72	17.28	0.0018	0.00204	<	4.65	6.75	21.93	4.21	4.81	13.19
6675.49	0.61	0.0018	0.00395	<	0.79	0.35	0.19	1.11	0.15	0.12
5107.76	0.6	0.0018	0.0415	<	1.06	0.68	0.46	0.75	0.39	0.21
1066.66	2.19	0.00235	0.00395	<	2.11	2.69	0.5	3.78	2.72	0.48
3451.96	4.43	0.00304	0.000502	0.0108	4.87	7.59	9.3	2.36	2.93	4.97
6961.16	0.94	0.00344	0.188	<	1.33	0.45	0.39	1.31	0.22	0.17

1082.58	1.23	0.0035	0.0188	<	1.46	1.77	0.54	1.68	1.78	0.4
3824.41	10.35	0.00362	0.00164	<	7.01	17.36	13.97	9.17	9.38	9
3926.79	3.07	0.00399	0.00146	8.7E-06	2.95	4.44	6.02	1.73	2.27	2.69
5214.05	0.78	0.00399	0.00267	<	1.07	0.37	0.28	1.04	0.15	0.23
1277.48	1.12	0.00402	0.0282	<	1.54	1.81	0.69	1.65	1.63	0.37
8923.81	0.37	0.00402	0.0044	<	0.46	0.14	0.08	0.6	0.06	0.07
1515.16	4	0.00425	0.00149	<	4.91	1.54	0.9	6.38	1.27	0.69
5539.07	0.47	0.00425	0.011	<	0.69	0.31	0.22	0.62	0.06	0.15
8309.05	0.16	0.00425	0.000532	<	0.16	0.29	0.32	0.11	0.16	0.17
5045.71	1.29	0.00444	0.0434	0.00999	2.56	2.39	1.27	1.62	1.15	0.86
5067.89	0.84	0.00549	0.0559	0.0426	1.7	1.32	0.86	1.09	0.61	0.46
9308.27	0.15	0.00563	0.0555	<	0.24	0.12	0.09	0.21	0.07	0.05
3944.7	1.91	0.00572	0.0044	0.0203	2.89	3.13	4.8	1.5	1.21	1.5
6101.78	0.36	0.00572	0.00599	4.59E-06	0.53	0.33	0.18	0.41	0.14	0.2
3966.99	0.95	0.00728	0.00808	0.255	1.69	1.52	2.47	0.84	0.56	0.78
6886.15	0.9	0.00728	0.0952	<	1.23	0.4	0.32	1.34	0.19	0.25
3744.32	15.85	0.00741	0.0122	<	10.42	25.32	9.48	14.17	18.55	6.15
3329.56	9.38	0.0094	0.142	<	17.8	13.69	8.41	25.14	7.28	2.77
6658.95	0.5	0.0094	0.0051	<	0.7	0.32	0.19	0.87	0.15	0.16
7844.58	0.31	0.0094	0.000855	<	0.21	0.4	0.52	0.17	0.35	0.33
6635.33	0.82	0.00984	0.0228	<	1.06	0.4	0.24	1.6	0.24	0.17
5195.8	0.87	0.012	0.00681	<	1.32	0.56	0.45	1.35	0.3	0.32
5027.1	1.49	0.0133	0.0412	0.00329	2.64	2.23	1.15	1.83	1.16	1.13
4986.66	2.08	0.017	0.011	0.000446	3.5	2.49	1.42	2.35	1.58	1.66
7910.2	0.28	0.0174	0.000427	<	0.18	0.46	0.35	0.14	0.62	0.2
1072.43	1.27	0.0211	0.0325	<	1.46	1.88	0.61	1.5	2.11	0.73
1078.05	0.84	0.0211	0.0646	<	1.51	1.31	0.67	1.18	1.32	0.64
5005.87	3.54	0.0215	0.0173	0.000133	6.01	4.34	2.47	4.05	2.79	2.99
4965.81	4.06	0.0297	0.0278	3.15E-05	6.94	4.59	2.88	5.11	3.42	3.58
6906.43	0.43	0.0332	0.467	<	0.75	0.36	0.32	0.8	0.24	0.18
1419.72	2.76	0.0525	0.046	<	3.58	1.14	0.82	5.65	1.24	0.91
1553.98	18.26	0.062	0.191	<	3.33	9.57	21.59	3.46	13	34.64
9154.71	0.1	0.0632	0.195	<	0.21	0.14	0.11	0.18	0.07	0.07
3443.74	3.3	0.0823	0.423	0.000765	10.21	10.97	7.67	8.38	5.93	2.41
1575.96	1.47	0.0854	0.338	<	0.99	2.46	2.22	0.56	2.95	3.13
3412.32	2.19	0.0889	0.0415	<	2.68	2.56	4.75	1.79	0.88	2.92
7777.74	0.38	0.0955	0.389	<	0.59	0.3	0.21	0.95	0.15	0.16
7568.07	2.66	0.103	0.00395	<	0.36	3.02	1.27	0.61	7.49	1.62
7585.65	2.08	0.108	0.00544	<	0.32	2.4	1.06	0.39	5.67	1.38
7667.06	0.36	0.116	0.0952	<	0.38	0.74	0.58	0.31	0.78	0.43
4940.04	0.68	0.141	0.0323	0.00483	1.54	1.22	0.87	0.79	0.85	1.03
1310.63	3.43	0.154	0.471	<	4.98	2.74	1.56	11.91	3.04	1.29
7938.7	0.66	0.16	0.163	<	0.19	0.85	0.33	0.13	1.9	0.3
1351.52	1.18	0.166	0.38	<	2.37	1.46	1.2	2.7	1.15	1.04
1466.81	2.89	0.171	0.567	<	5.89	3	3.22	7.2	2.81	3.19
2234	2	0.171	0.0154	<	3.45	3.73	1.74	2.93	4.95	2.47
5900.78	0.78	0.171	0.0952	<	0.72	0.58	1.36	0.38	0.21	2.09
7954.23	0.55	0.198	0.133	<	0.21	0.76	0.32	0.15	1.57	0.27
7517.02	0.53	0.198	0.337	<	0.25	0.47	0.78	0.15	0.68	1.31
3880.01	1.24	0.208	0.0608	<	2.39	2.92	3.62	1.81	1.46	2
4420.12	1.44	0.217	0.0703	<	2.42	1.22	0.98	3.78	0.77	1.26
5080.45	0.6	0.232	0.37	<	1.97	1.4	1.37	1.53	0.96	0.85
3277.09	2.71	0.27	0.0868	8.68E-05	6.4	8.3	9.11	6.17	4.07	4.16
7831.65	0.15	0.317	0.0243	<	0.35	0.48	0.5	0.39	0.49	0.22
4588.12	0.26	0.359	0.193	6.62E-05	0.81	0.94	1.07	0.49	0.29	0.57
7497.83	0.53	0.453	0.484	<	0.36	0.45	0.89	0.23	0.52	1.47
1617.8	1.09	0.475	0.0236	<	2.54	1.54	1.45	3.82	2.44	1.96
1537.9	4.17	0.498	0.169	<	4.09	8.25	7.57	7.52	16.73	12.95
5939.83	0.08	0.498	0.0738	3.35E-05	0.49	0.42	0.48	0.29	0.16	0.71
6721.15	0.13	0.527	0.791	<	0.54	0.47	0.41	0.47	0.32	0.24
5159.91	0.17	0.549	0.665	1.47E-06	0.78	0.61	0.71	0.65	0.42	0.45
2380.92	1.21	0.659	0.118	1.48E-06	3.14	3.81	2.6	1.72	3.52	3.31
5869.32	0.26	0.688	0.714	<	0.56	0.53	0.79	0.37	0.24	0.93
2628.53	2.09	0.71	0.0758	<	5.93	5.09	3.83	9.2	3.92	5.73
7438.93	0.05	0.738	0.445	<	0.27	0.24	0.22	0.22	0.06	0.16
8581.07	0.18	0.762	0.425	8.59E-05	0.78	0.72	0.6	0.64	0.5	0.69
2682.9	1.25	0.774	0.0415	<	3.13	3.98	2.73	2.29	5.5	4.34
7748.31	0.07	0.774	0.373	7.12E-05	0.4	0.43	0.36	0.35	0.2	0.33

2325.37	0.6	0.932	0.337	<	3.66	3.93	3.33	4.34	4.61	4.15
8446.32	0.02	0.932	0.233	<	0.15	0.15	0.17	0.1	0.06	0.2
8486.64	0.01	0.932	0.264	0.000622	0.16	0.17	0.18	0.1	0.06	0.2

Table SM 13. The discriminant masses established for the Poor Outcome SAH patients for the samples collected at days 1, 5 and 10 after the SAH incident.

**Genetic Algorithm (GA)**			**Quick Classifier (QC)**			**Supervised Neural Network (SNN)**		
**Mass (m/z)**	*Start Mass*	*End mass*	**Mass (m/z)**	Start Mass	End mass	**Mass (m/z)**	Start Mass	End mass
3587.39	*3571.12*	*3597.45*	1866.52	*1861.04*	*1875.61*	1553.98	*1550.06*	*1561.5*
4568.69	*4558.85*	*4578.27*	2022.82	*2017.69*	*2028.62*			
6520.37	*6504.14*	*6533.85*	2044.66	*2039.42*	*2050.25*			
5027.1	*5018.17*	*5037.01*	2580.47	*2574.26*	*2592.9*			
3966.99	*3959.74*	*3977.84*	2755.51	*2744.22*	*2765.57*			
8309.05	*8276.04*	*8319.24*	2939.41	*2935.76*	*2947.19*			
3539.15	*3531.71*	*3549.66*	3350.84	*3341.94*	*3362.34*			
3517.15	*3508.64*	*3531.71*	3371.57	*3362.34*	*3382.6*			
3784.21	*3774.84*	*3797.43*	3476.86	*3465.09*	*3485.86*			
1351.52	*1347.27*	*1360.2*	3498.42	*3492.95*	*3506.71*			
			3517.15	*3508.64*	*3531.71*			
			3539.15	*3531.71*	*3549.66*			
			3587.39	*3571.12*	*3597.45*			
			3765.82	*3759.23*	*3774.84*			
			3904.37	*3890.05*	*3915.72*			
			4054.12	*4045.53*	*4065.44*			
			6520.37	*6504.14*	*6533.85*			
			6807.14	*6768.6*	*6822.98*			
			6846.72	*6836.83*	*6862.77*			

PART III.

Table SM 14. Comparison of CSF samples derived from Poor Outcome SAH (PO-SAH) and Good Outcome SAH (GO-SAH) patients. Samples collected at day 1 of SAH incident.

Abbreviations: Mass (m/z) - mass of the selected peaks; **DAve** – difference between the maximal and minimal average peak intensity of all classes; **PTTA** – p-value of t-test (2 classes) or ANOVA test (>2 classes), range 0-1, where: 0-good, 1-bad, preferable for normal distributed data; **PWKW** - p-value of Wilcoxon test (2 classes) or Kruskal-Wallis test (>2 classes), range 0-1, where: 0-good, 1-bad, preferable for not normal distributed data; **PAD** - p-value of Anderson-Darling test, gives information about normal distribution, range 0-1, where: 0-not normal distributed, 1-normal distributed data, "<" p-value less than 0.000001 ; **PO-Day1, GO-Day1** – average peak intensity of classes PO-SAH Day1 and GO-Day1; **SD PO-Day**, **SD GO-Day** – standard deviation of the peak intensity average of classes PO-SAH Day1 and GO-Day1.

Mass (m/z)		DAve	PTTA	PWKW	PAD	PO-Day1	GO-Day1	SD PO-Day1	SD GO-Day1
1050.2		1.09	0.0393	0.00394	<	1.07	2.16	1.3	1.35
1066.59		1.25	0.418	0.0407	<	2.11	3.36	3.78	4.18
1072.38		1.83	0.1	0.0429	<	1.46	3.29	1.5	3.43
1078.03		1.65	0.0935	0.0622	<	1.57	3.22	1.22	3.02
1082.15		0.99	0.142	0.0427	<	1.41	2.4	1.68	2.02
1088.54		1.23	0.107	0.0521	<	1.25	2.48	1.09	2.36
1094.24		1.5	0.0469	0.0407	<	0.99	2.49	0.75	2.36
1207.14		1.47	0.161	0.4	<	1.76	3.23	1.7	3.52
1261.27		2.89	0.0788	0.0789	<	1.62	4.51	1.27	5.1
1277.48		1.5	0.118	0.0169	<	1.55	3.04	1.65	2.93
1283.23		1.72	0.0408	0.00808	<	1	2.72	0.87	2.6
1310.13		3.32	0.526	0.0429	<	4.97	8.29	11.92	14.84
1332.68		2.14	0.109	0.234	<	1.6	3.74	1.7	4.22
1351.32		0.49	0.64	0.38	<	2.37	2.86	2.7	2.74
1419.78		0.73	0.708	0.926	<	3.74	4.47	5.57	5.18
1442.26		0.17	0.769	0.93	<	2.14	1.97	1.98	1.46
1466.65		2.79	0.491	0.971	<	5.89	8.68	7.2	11.94
1488.69		1.44	0.226	0.234	<	1.63	3.07	1.53	4.04
1510.45		0.85	0.267	0.797	<	1.61	2.46	1.11	2.58
1515.09		2.26	0.142	0.837	<	4.88	2.61	6.39	1.5
1537.95		2.1	0.246	0.99	<	4.08	1.98	7.53	1.1
1553.92		1.71	0.418	0.102	<	3.33	5.03	3.46	6.61
1617.71		1.39	0.135	0.949	<	2.52	1.14	3.83	0.71
1779.39		0.87	0.123	0.989	<	2.27	1.4	2.2	0.67
1796.5		0.08	0.891	0.589	<	2.11	2.19	1.67	1.97
1866.54		3.37	0.0935	0.323	<	6.57	3.19	7.14	2.7
1888.67		0.25	0.715	0.181	2.77E-05	2.35	1.23	2.35	47.29
2022.73		9.1	0.0788	0.0716	<	19.81	10.71	15.59	10.93
2033.36		3.64	0.000948	0.000397	<	5.15	1.5	3.92	0.81
2044.5		1.34	0.105	0.171	<	3.74	2.4	2.5	1.83
2380.08		0.37	0.684	0.921	<	3.15	3.52	1.71	2.72
2424.18		1.94	0.618	0.99	<	7.67	9.61	7.09	11.77
2580.26		1.62	0.598	0.837	<	10.11	8.49	10.18	5.81
2755.27		16.45	0.134	0.493	<	36.35	19.9	40.58	19.41
2777.15		2.7	0.231	0.498	<	7.12	4.42	8.39	4.01
2795.1		5.85	0.123	0.384	<	12.01	6.16	14.67	5.01
2884.74		1.58	0.598	0.279	<	4.64	6.22	3.11	9.68
2939.1		9.28	0.0935	0.27	<	19.05	9.77	19	8.75
3201.05		5.12	0.0141	0.0407	<	6.58	1.46	7.31	1.47
3277.08		1.99	0.419	0.219	<	6.4	8.39	6.16	6.76
3329.35		5.07	0.506	0.897	<	17.8	12.73	25.14	13.34
3350.49		1.16	0.506	0.358	<	5.51	4.35	5.14	3.84

3371.08		3.47	0.123	0.608	3.59E-06	10.32	6.85	8.32	3.53
3390.7		0.71	0.68	0.797	<	4.59	5.3	4.25	4.58
3430.45		5.44	0.231	0.99	<	9	14.44	6.17	15.48
3443.62		3.29	0.127	0.57	3.51E-05	10.29	7	8.34	2.9
3476.44		36.94	0.132	0.484	<	28.63	65.57	27.27	83.21
3497.78		4.52	0.158	0.179	<	6.62	11.14	6.26	10.33
3515.91		6.93	0.1	0.149	<	8.33	15.26	6.29	12.75
3538.16		1.54	0.1	0.0584	<	2.62	4.15	2.03	2.62
3743.81		6.75	0.466	0.438	<	10.2	16.95	14.28	28.55
3765.46		1.14	0.519	0.0549	<	4.67	3.54	3.31	5.19
3783.49		0.01	0.995	0.301	<	3.49	3.48	4.04	5.2
3823.91		0.61	0.848	0.791	<	7.02	7.63	9.17	10.23
3845.43		0.25	0.726	0.641	<	2.04	2.3	2.09	2.23
3857.34		2.93	0.215	0.971	<	3.03	5.96	2.52	8.13
3903.92		5.05	0.0408	0.0431	<	3.04	8.08	2.3	7.7
3926.46		1.17	0.142	0.234	0.000588	2.95	4.12	1.73	2.51
3943.72		1.2	0.0788	0.0584	0.0598	2.56	3.76	1.49	1.85
3966.4		0.66	0.0292	0.024	0.468	1.62	2.28	0.83	0.62
4054.16		1.87	0.109	0.0407	<	3.94	2.07	3.58	2.64
4210.68		0.59	0.529	0.901	<	2.06	1.47	2.78	2.28
4419.57		0.66	0.526	0.797	<	2.42	1.76	3.78	1.65
4587.8		0.41	0.0334	0.0169	0.269	0.81	1.22	0.49	0.46
4818.1		0.74	0.343	0.467	<	0.65	1.39	0.32	2.66
4938.86		0.32	0.344	0.162	0.00411	1.53	1.21	0.79	0.96
4964.42		2.37	0.161	0.171	3.99E-05	6.94	4.57	5.11	4.5
4985.72		0.82	0.365	0.372	0.000227	3.5	2.68	2.35	2.45
5004.22		1.73	0.259	0.234	1.78E-05	6.01	4.28	4.05	4.39
5025.72		0.44	0.553	0.503	2.18E-05	2.64	2.2	1.83	2.05
5043.78		0.11	0.872	0.971	0.000329	2.56	2.67	1.62	2.28
5065.82		0.14	0.718	0.837	0.00312	1.72	1.86	1.08	1.23
5083.62		0.69	0.118	0.234	3.02E-05	1.96	1.27	1.53	0.81
5106.53		0.07	0.806	0.837	0.000276	1.04	0.98	0.75	0.84
5127.82		0.24	0.259	0.681	0.00174	0.96	0.71	0.74	0.46
5155.76		0.2	0.64	0.99	<	0.79	0.99	0.64	1.38
5193.75		0.8	0.0334	0.0169	<	1.3	0.51	1.35	0.3
5213.08		0.64	0.0292	0.126	<	1.07	0.42	1.04	0.2
5455.72		0.11	0.667	0.832	<	0.55	0.65	0.48	0.73
5537.36		0.22	0.365	0.276	4.29E-05	0.69	0.91	0.62	0.66
5577.79		0.06	0.708	0.99	1.02E-06	0.57	0.63	0.4	0.49
5867.93		0.71	0.00861	0.00244	1.58E-05	0.59	1.3	0.37	0.75
5889.11		0.48	0.0334	0.0407	0.00206	0.71	1.19	0.38	0.64
6172.3		0.05	0.598	0.791	0.0577	0.48	0.43	0.3	0.21
6434.75		0.06	0.715	0.75	<	0.59	0.52	0.52	0.45
6518.61		0.11	0.718	0.877	<	1.04	0.93	0.89	0.91
6633.02		0.31	0.524	0.797	<	1.06	0.75	1.6	0.87
6656.27		0.02	0.926	0.994	<	0.69	0.67	0.87	0.61
6673.28		0.06	0.838	0.99	<	0.8	0.73	1.11	0.75
6695.45		0.08	0.64	0.647	<	0.47	0.56	0.52	0.42
6717.84		0.14	0.37	0.234	1.29E-05	0.53	0.68	0.47	0.41
6805.37		3.73	0.142	0.57	<	8.45	4.72	8.95	5.32
6844.92		0.84	0.37	0.971	<	3.01	2.17	3.01	2.09
6884.35		0.16	0.685	0.857	7.27E-06	1.23	1.06	1.34	0.84
6937.45		0.38	0.519	0.489	<	1.03	1.41	1.18	1.7
6960.04		0.1	0.823	0.73	<	1.33	1.44	1.31	1.41
7480.41		0.63	0.118	0.0622	<	0.37	1	0.24	1.35
7557.61		5.98	0.195	0.0169	<	0.35	6.33	0.61	16.43
7575.9		4.08	0.191	0.0348	<	0.35	4.44	0.5	11.07
7661.03		0.7	0.0393	0.00438	<	0.38	1.08	0.31	1.04
7776.34		0.15	0.526	0.276	<	0.61	0.46	0.94	0.21
7821.96		0.2	0.132	0.0321	1.07E-06	0.35	0.55	0.39	0.34
7927.13		0.6	0.123	0.00453	<	0.18	0.78	0.12	1.33
7947.19		0.49	0.118	0.00808	<	0.2	0.69	0.14	1.05
8301.53		0.25	0.0788	0.0075	<	0.15	0.4	0.11	0.44
8557.37		0.29	0.215	0.38	<	0.48	0.77	0.42	0.76
8575.32		0.06	0.708	0.971	0.0148	0.7	0.64	0.54	0.34
8595.29		0.08	0.692	0.508	0.000236	0.71	0.79	0.62	0.5
8924.07		0.16	0.343	0.99	<	0.46	0.3	0.6	0.26

Table SM 15. The discriminant masses between Poor Outcome SAH (PO-SAH) and Good Outcome SAH (GO-SAH) for cerebrospinal fluid samples collected at day 1 of SAH incident.

**Genetic Algorithm (GA)**			**Quick Classifier (QC)**			**Supervised Neural Network (SNN)**		
**Mass (m/z)**	*Start Mass*	*End mass*	**Mass (m/z)**	Start Mass	End mass	**Mass (m/z)**	Start Mass	End mass
4938.86	*4903.06*	*4944.41*	1050.2	*1046.49*	*1058.12*	3371.08	*3361.27*	*3383*
1553.92	*1549.88*	*1561.04*	1094.24	*1091.49*	*1097.85*	1072.38	*1069.82*	*1075.28*
1207.14	*1203.01*	*1210.68*	1261.27	*1256.11*	*1270.02*	1094.24	*1091.49*	*1097.85*
7821.96	*7796.55*	*7835.86*	1283.23	*1280.63*	*1286.47*	4587.8	*4579.58*	*4597.56*
2044.5	*2039.06*	*2050.87*	2022.73	*2017.66*	*2028.75*	1888.67	*1879.98*	*1894.79*
3966.4	*3960.93*	*3976.73*	2033.36	*2029.57*	*2039.06*	4938.86	*4903.06*	*4944.41*
1796.5	*1790.66*	*1807.89*	3201.05	*3193.48*	*3212.19*	5193.75	*5181.21*	*5202.97*
3443.62	*3437.09*	*3457.35*	3903.92	*3889.44*	*3915.55*	2044.5	*2039.06*	*2050.87*
5025.72	*5017.05*	*5034.85*	3943.72	*3935.35*	*3946.08*	1283.23	*1280.63*	*1286.47*
2795.1	*2785.16*	*2808.58*	3966.4	*3960.93*	*3976.73*	3903.92	*3889.44*	*3915.55*
			4587.8	*4579.58*	*4597.56*	2777.15	*2772.15*	*2785.16*
			5193.75	*5181.21*	*5202.97*	3201.05	*3193.48*	*3212.19*
			5213.08	*5202.97*	*5226.35*	1078.03	*1075.28*	*1080.63*
			5867.93	*5819.71*	*5880.4*	7947.19	*7938.46*	*7954.06*
			5889.11	*5880.4*	*5926.82*	8557.37	*8511.96*	*8563.16*
			7661.03	*7652.75*	*7695.2*	5867.93	*5819.71*	*5880.4*
			8301.53	*8291.42*	*8316*	7927.13	*7915.42*	*7934.56*
						1261.27	*1256.11*	*1270.02*
						4818.1	*4796.94*	*4827.21*
						2795.1	*2785.16*	*2808.58*
						7480.41	*7452.93*	*7494.2*

PART IV.

Table SM 16. Comparison of CSF samples derived from Poor Outcome SAH (PO-SAH) and Good Outcome SAH (GO-SAH) patients. Samples collected at day 5 of SAH incident.

Abbreviations: Mass (m/z) - mass of the selected peaks; **DAve** – difference between the maximal and minimal average peak intensity of all classes; **PTTA** – p-value of t-test (2 classes) or ANOVA test (>2 classes), range 0-1, where: 0-good, 1-bad, preferable for normal distributed data; **PWKW** - p-value of Wilcoxon test (2 classes) or Kruskal-Wallis test (>2 classes), range 0-1, where: 0-good, 1-bad, preferable for not normal distributed data; **PAD** - p-value of Anderson-Darling test, gives information about normal distribution, range 0-1, where: 0-not normal distributed, 1-normal distributed data, "<"p-value less than 0.000001 ; **PO-Day5, GO-Day5** – average peak intensity of classes PO-SAH Day5 and GO-Day5; **SD PO-Day**, **SD GO-Day** – standard deviation of the peak intensity average of classes PO-SAH Day5 and GO-Day5.

Mass (m/z)	DAve	PTTA	PWKW	PAD	PO-Day5	GO-Day5	SD PO-Day5	SD GO-Day5
2755.26	13.34	0.182	0.0752	<	16.38	3.03	21.07	2.26
3276.69	8.83	0.182	0.371	1.32E-06	17.11	4.09	13.94	49.4
3390.46	5.38	0.182	0.298	3.16E-06	12.12	4.81	8.65	71.38
2795.06	2.97	0.182	0.0953	<	4.91	1.94	4.87	0.77
1332.38	1.83	0.182	0.236	<	1.14	2.97	0.77	3.03
5866.11	0.53	0.182	0.395	<	0.53	1.06	0.23	0.96
2031.65	2.08	0.189	0.236	<	3.41	1.33	3.41	1.47
3965.99	0.51	0.189	0.298	0.0135	2.03	0.56	0.84	36.61
2939.02	5.41	0.193	0.118	<	8.25	2.83	9.75	1.69
3297.6	2.12	0.193	0.298	<	2.02	4.15	1.26	4.28
2777.06	3.3	0.195	0.298	<	4.25	0.95	6.32	0.46
3317.43	3.23	0.195	0.298	<	2.35	5.58	0.77	7.04
3411.76	1.95	0.195	0.527	<	2.56	4.51	0.89	4.14
5886.96	0.34	0.212	0.994	<	0.52	0.86	0.19	0.76
4419.35	0.47	0.228	0.298	5.98E-06	0.74	0.77	0.72	63.24
2324.75	2.2	0.246	0.298	<	3.93	1.73	4.61	1.46
3845.7	1.4	0.246	0.298	0.0362	3.37	2.26	2.37	47.41
6938.43	0.24	0.246	0.648	<	0.33	0.57	0.18	0.57
1309.95	6.87	0.258	0.341	<	2.75	9.62	3.04	17.23
3879.51	0.75	0.277	0.349	0.0079	2.17	1.46	1.1	50.04
3328.92	19.64	0.28	0.648	<	13.69	33.34	7.28	54.4
1537.54	6.93	0.28	0.566	<	8.25	1.32	16.73	0.97
5005.03	1.31	0.28	0.371	0.141	3.02	2.79	2.06	64.26
3944.14	0.97	0.28	0.444	0.000237	4.09	1.21	2.26	38.85
2913	0.73	0.28	0.371	0.0141	2.81	1.35	1.45	38.19
5026.84	0.6	0.28	0.395	0.0372	1.62	1.17	0.96	52.6
4607.58	0.18	0.28	0.515	0.0046	0.88	0.24	0.42	33.91
3350.5	5.94	0.287	0.745	<	3.53	9.48	1.58	17.3
1553.79	5.24	0.287	0.535	<	9.57	4.33	13	3.86
3370.87	6.73	0.292	0.994	<	8.75	15.48	5.45	19.29
2628.86	1.75	0.292	0.515	1.43E-05	3.34	3.92	2.91	76.94
3587.14	2.18	0.322	0.745	<	4.5	6.68	2.42	6.45
7487.78	1.03	0.322	0.395	<	0.45	1.48	0.52	3.28
3806.35	0.56	0.322	0.349	0.000501	1.59	1.19	1.19	55.2
6635.42	0.09	0.322	0.645	0.00209	0.31	0.24	0.13	58.72
5077.49	0.38	0.344	0.466	<	1.39	1.01	0.96	0.63
3823.9	4.27	0.388	0.44	0.0131	13.09	9.38	11.55	54.03
3903.67	3.19	0.388	0.56	0.000114	12.06	6.06	9.57	68.34
4123.75	1.44	0.388	0.515	<	2.46	1.02	4.66	0.72
4964.95	1.26	0.388	0.527	0.0098	3.33	3.42	2.35	74.58
7507.39	0.74	0.388	0.44	<	0.44	1.19	0.58	2.56
4516.66	0.61	0.388	0.515	<	1.24	0.63	1.97	0.29

8605.98	0.13	0.388	0.515	0.000874	0.43	0.36	0.29	63.59
4252.12	0.7	0.407	0.745	<	1.49	0.8	2.31	0.51
3765.47	1.57	0.415	0.298	6.44E-06	4.05	4.02	4.37	71.49
4986.77	0.56	0.415	0.722	0.0249	1.93	1.58	1.34	63.65
6958.05	0.13	0.415	0.986	<	0.42	0.55	0.21	0.47
2580.25	2.7	0.415	0.986	<	3.33	6.03	3.14	10.89
3783.8	1.38	0.415	0.298	1.36E-05	3.96	3.61	3.92	67.49
1077.92	0.67	0.415	0.796	<	1.3	1.98	1.33	2.34
4587.76	0.14	0.415	0.638	0.0342	1.09	0.29	0.48	31.03
6883.83	0.13	0.415	0.949	<	0.4	0.53	0.19	0.51
6657.79	0.05	0.415	0.528	0.00116	0.27	0.15	0.13	45.9
2380.49	0.89	0.544	0.918	8.3E-06	2.92	3.52	2.02	92.45
1094.09	0.49	0.559	0.949	<	1.3	1.8	1.39	2.02
2044.49	1.57	0.576	0.986	<	3.7	2.13	7.04	2.46
5044.46	0.38	0.576	0.515	0.0306	2.02	1.15	1.58	48.2
3556.8	0.57	0.6	0.796	0.174	4.48	1.93	2.44	49.36
3857.16	2.11	0.601	0.804	4.14E-05	8.64	9.09	7.26	84.46
3538.32	1.08	0.601	0.82	0.438	8.42	3.93	4.69	53.55
3926.08	0.73	0.601	0.949	0.000318	5.17	2.27	3.62	51.2
1066.52	0.66	0.601	0.638	<	2.7	2.04	2.72	2.53
8299.88	0.06	0.601	0.949	<	0.29	0.36	0.16	0.32
4938.57	0.19	0.615	0.694	0.0147	1.03	0.85	0.67	69.97
3609.05	0.52	0.643	0.949	2.71E-06	3.14	1.87	2.72	71.43
3476.32	19.02	0.644	0.949	0.00564	130.17	73.78	101.68	66.38
3516.38	3.27	0.644	0.796	0.0258	28.42	14.68	15.71	58.41
2342.42	0.52	0.644	0.994	<	2.73	3.25	2.22	2.62
3443.27	2.3	0.678	0.433	<	11.02	13.32	5.91	15.85
1739	0.32	0.682	0.949	0.0327	2.99	1.31	1.94	49
5157.01	0.11	0.692	0.528	<	0.64	0.75	0.4	0.74
6675.88	0.03	0.692	0.528	5.98E-06	0.32	0.15	0.18	42.53
3743.86	3.78	0.694	0.89	8.23E-05	21.54	18.55	23.14	73.25
3497.83	2.18	0.703	0.89	0.667	24.25	11.83	13.94	53.6
5108.16	0.07	0.703	0.868	2.23E-05	0.61	0.39	0.41	57.78
5937.42	0.04	0.703	0.349	0.0046	0.37	0.16	0.3	39.43
8570.11	0.08	0.717	0.89	0.000744	0.64	0.5	0.49	70.57
6518.19	0.04	0.717	0.949	<	0.31	0.35	0.19	0.36
3430.35	1.92	0.73	0.949	0.00776	22.58	13.87	13.21	67.16
7954.65	0.25	0.73	0.0953	<	0.77	1.01	1.57	1.84
6866.69	0.06	0.73	0.949	<	0.54	0.48	0.42	0.34
6720.13	0.04	0.73	0.949	1.22E-06	0.43	0.32	0.19	68.69
5066.7	0.1	0.731	0.648	0.0163	1.23	0.62	0.87	46.65
7937.05	0.24	0.786	0.371	<	0.88	1.12	1.88	2.27
7566.04	0.81	0.804	0.371	<	3.02	3.83	7.49	8.4
1088.39	0.19	0.804	0.949	<	1.74	1.93	1.78	1.89
2022.58	1.99	0.827	1	<	13.71	11.72	25.78	17.7
1466.61	0.22	0.827	0.994	2.44E-05	2.78	2.81	2.02	93.83
6805.37	0.14	0.851	0.949	<	1.43	1.29	1.79	2.1
6845.51	0.05	0.851	0.794	<	0.67	0.72	0.57	0.65
7584.21	0.33	0.875	0.349	<	2.4	2.73	5.67	6.03
1082.4	0.1	0.875	0.949	<	1.77	1.87	1.78	1.91
4567.67	0.07	0.892	0.812	0.000955	2.42	1.57	1.53	66.67
7833.74	0.02	0.892	0.371	<	0.49	0.51	0.49	0.26
1072.25	NaN	1	0.949	<	1.88	1.88	2.11	2.48

Table SM 17. The discriminant masses between Poor Outcome SAH (PO-SAH) and Good Outcome SAH (GO-SAH) for cerebrospinal fluid samples collected at day 5 of SAH incident.

**Genetic Algorithm (GA)**			**Quick Classifier (QC)**			**Supervised Neural Network (SNN)**		
**Mass (m/z)**	*Start Mass*	*End mass*	**Mass (m/z)**	Start Mass	End mass	**Mass (m/z)**	Start Mass	End mass
2628.86	*2616.64*	*2637.29*	1332.38	*1325.72*	*1340.94*	5077.49	*5073.5*	*5101.79*
3823.9	*3814.85*	*3834.86*	2031.65	*2029*	*2039.3*	4252.12	*4241.3*	*4264.06*
4938.57	*4916.4*	*4945.27*	**2755.26**	*2744.43*	*2766.73*	**6866.69**	*6860.34*	*6876.35*
**3276.69**	*3268.2*	*3290.87*	**2795.06**	*2784.52*	*2807.56*	3443.27	*3437.09*	*3449.67*
1077.92	*1075.03*	*1080.03*	**3276.69**	*3268.2*	*3290.87*	**2755.26**	*2744.43*	*2766.73*
8605.98	*8599.01*	*8694.33*	**3390.46**	*3382.78*	*3402.67*	4123.75	*4113.24*	*4130.74*
1088.39	*1085.75*	*1091.61*	5866.11	*5841.49*	*5873.54*	**2795.06**	*2784.52*	*2807.56*
**3390.46**	*3382.78*	*3402.67*				7954.65	*7943.71*	*7973.32*
**6866.69**	*6860.34*	*6876.35*				7937.05	*7904.76*	*7943.71*
5108.16	*5101.79*	*5126.25*				1309.95	*1306.53*	*1319.53*
						7584.21	*7579.18*	*7633.86*
						1537.54	*1533.17*	*1545.12*
						7566.04	*7531.96*	*7574.1*
						3317.43	*3305.06*	*3320.95*
						2777.06	*2770.93*	*2784.52*
						3350.5	*3342.13*	*3357.9*
						3297.6	*3290.87*	*3305.06*
						1066.52	*1062.96*	*1069.7*
						2324.75	*2311.78*	*2336.27*
						**3276.69**	*3268.2*	*3290.87*
						3411.76	*3405.01*	*3418.59*

PART V.

Table SM 18. Comparison of CSF samples derived from Poor Outcome SAH (PO-SAH) and Good Outcome SAH (GO-SAH) patients. Samples collected at day 10 of SAH incident.

Abbreviations: Mass (m/z) - mass of the selected peaks; **DAve** – difference between the maximal and minimal average peak intensity of all classes; **PTTA** – p-value of t-test (2 classes) or ANOVA test (>2 classes), range 0-1, where: 0-good, 1-bad, preferable for normal distributed data; **PWKW** - p-value of Wilcoxon test (2 classes) or Kruskal-Wallis test (>2 classes), range 0-1, where: 0-good, 1-bad, preferable for not normal distributed data; **PAD** - p-value of Anderson-Darling test, gives information about normal distribution, range 0-1, where: 0-not normal distributed, 1-normal distributed data, "<" p-value less than 0.000001 ; **PO-Day10, GO-Day10** – average peak intensity of classes PO-SAH Day10 and GO-Day10; **SD PO-Day**, **SD GO-Day** – standard deviation of the peak intensity average of classes PO-SAH Day10 and GO-Day10.

Mass (m/z)	DAve	PTTA	PWKW	PAD	PO-Day10	GO-Day10	SD PO-Day10	SD GO-Day10
2428.48	2.53	0.0538	0.0931	<	1.23	3.75	1.15	3.38
4250.92	1.81	0.0538	0.0931	<	0.53	2.33	0.3	2.38
1345.46	1.1	0.0538	0.0931	0.000082	1.03	2.13	0.73	1.36
7731.52	0.33	0.0538	0.0931	0.000787	0.27	0.6	0.22	0.39
7789.55	0.25	0.0538	0.0931	0.000922	0.21	0.46	0.16	0.32
1332.57	2.04	0.0578	0.0931	<	0.61	2.65	0.31	3.29
1247.71	1.35	0.0578	0.0931	<	1.03	2.38	0.74	2.07
7665.77	0.66	0.0578	0.12	5.66E-05	0.56	1.22	0.42	0.97
5109.08	0.32	0.0578	0.116	0.000165	0.46	0.77	0.21	0.45
7886.49	0.28	0.0578	0.116	0.00343	0.34	0.62	0.17	0.4
6864.3	0.2	0.0578	0.0931	<	0.27	0.47	0.08	0.33
6679.26	0.19	0.0604	0.0931	4.09E-05	0.18	0.37	0.12	0.28
1509.14	1.93	0.0631	0.116	<	0.79	2.72	0.55	3.34
2342	2.18	0.0767	0.0931	0.000045	1.8	3.98	1.72	3.24
6461.82	0.35	0.0783	0.0931	<	0.15	0.49	0.11	0.63
1288.84	1.8	0.0786	0.0931	<	0.64	2.45	0.42	3.4
7829.03	0.27	0.0786	0.23	0.0115	0.5	0.76	0.22	0.4
3328.66	4.05	0.0849	0.261	0.000543	8.41	12.46	2.77	7.13
1266.61	1.3	0.0849	0.15	<	0.91	2.21	0.57	2.5
2912.68	1.27	0.0849	0.0931	5.93E-05	3.91	2.64	1.56	0.71
7910.02	0.47	0.0849	0.197	<	0.35	0.82	0.2	0.88
6256.4	0.14	0.0849	0.0931	2.35E-05	0.15	0.29	0.13	0.22
1488.5	2.04	0.0885	0.116	<	0.92	2.96	0.51	4.17
3370.84	1.56	0.0885	0.261	0.000187	3.51	5.08	0.98	2.95
3965.94	0.69	0.0885	0.116	0.303	2.47	1.78	0.78	0.87
3389.93	10.05	0.109	0.226	0.00015	21.93	11.88	13.19	8.07
7746.66	0.31	0.12	0.168	0.000537	0.35	0.67	0.34	0.51
8305.83	0.16	0.12	0.197	0.00353	0.34	0.5	0.17	0.26
3764.71	0.96	0.133	0.433	1.83E-06	1.57	2.53	0.81	1.94
7567.66	3.44	0.146	0.23	<	1.27	4.71	1.67	7.92
3783.38	1.03	0.173	0.727	<	1.55	2.57	0.46	2.5
3516.49	21.13	0.178	0.216	0.00343	56.62	35.48	32.57	21.56
5044.43	0.73	0.178	0.216	0.00041	1.27	2	0.86	1.42
6635.27	0.13	0.193	0.175	0.00185	0.24	0.37	0.17	0.25
7936.98	1.43	0.2	0.0931	<	0.34	1.77	0.31	3.81
2477.75	1.11	0.265	0.216	<	1.58	2.7	1.29	2.7
3476.11	69.47	0.271	0.297	0.000345	217.15	147.68	125.86	96.95
7489	3.03	0.271	0.15	<	0.74	3.77	1.1	9.39
2297.48	2.87	0.271	0.747	<	1.42	4.29	0.46	8.62
7507.16	2.03	0.271	0.197	<	0.78	2.81	1.31	5.89
1738.67	1.33	0.271	0.261	0.00922	4.53	3.2	2.5	1.62

1929.39	0.57	0.271	0.205	0.013	1.81	2.38	1.03	0.93
8577.2	0.39	0.271	0.129	0.0129	0.6	0.99	0.69	0.65
5157.67	0.33	0.271	0.409	<	0.71	1.04	0.45	0.8
3538.47	5.68	0.271	0.29	0.0653	16.07	10.38	11.05	6.51
1553.77	16.69	0.274	0.607	<	21.59	4.9	34.64	5.73
4372.03	3.05	0.274	0.124	<	0.69	3.74	1.06	9.79
6944.66	0.11	0.274	0.726	<	0.31	0.42	0.12	0.31
3856.91	5.63	0.287	0.358	0.00215	16.51	10.88	11.56	7.51
3557.31	2.56	0.287	0.216	0.198	8.04	5.48	5.41	3.2
3453.05	2.39	0.287	0.297	4.28E-05	9.26	6.87	4.82	3.83
3943.89	0.96	0.287	0.272	0.207	4.8	3.84	1.5	2.37
6885.44	0.2	0.287	0.261	<	0.32	0.52	0.25	0.56
3700.26	1.19	0.287	0.607	2.03E-05	3.57	2.38	2.6	1.1
3430.04	7.46	0.296	0.35	0.0172	25.11	17.66	15.82	11.05
3411.81	1.34	0.302	0.304	0.0192	4.75	3.41	2.92	1.87
1537.66	5.51	0.312	0.745	<	7.56	2.05	12.96	3.67
3296.48	0.49	0.318	0.29	0.303	1.96	2.45	0.96	1.12
3497.72	14.97	0.355	0.409	0.0192	48.36	33.39	36.18	22.1
3743.41	3.35	0.357	0.747	0.000398	9.48	12.82	6.15	9.89
3878.72	0.84	0.357	0.261	0.00343	3.62	2.78	2	1.58
6721.62	0.1	0.357	0.235	0.021	0.41	0.51	0.24	0.22
3608.87	0.82	0.367	0.609	1.38E-05	2.54	3.36	1.43	2.65
3586.86	1.63	0.378	0.727	1.58E-06	5.99	7.62	2.92	5.47
5867.03	0.39	0.378	0.15	0.000127	0.79	1.18	0.93	0.92
3903.43	3.14	0.378	0.342	0.0188	14.58	11.43	7.98	6.27
1749.32	0.3	0.38	0.367	0.0406	1.93	1.64	0.79	0.48
7843.84	0.13	0.405	0.4	0.0157	0.51	0.65	0.33	0.35
5897.83	0.69	0.418	0.245	<	1.35	0.66	2.1	0.47
4410.75	0.8	0.426	0.756	<	0.8	1.6	1.01	3.49
4987.83	0.61	0.461	0.216	<	1.42	2.03	1.66	1.87
4567.44	0.49	0.461	0.448	0.00384	3.22	2.73	1.47	1.23
6957.02	0.07	0.483	0.609	0.000449	0.37	0.44	0.18	0.25
5026.25	0.38	0.489	0.342	1.34E-05	1.14	1.52	1.13	1.15
3925.74	0.9	0.494	0.501	0.279	6.02	5.12	2.7	2.95
1707.07	0.5	0.524	0.284	<	2.24	2.74	0.62	2.77
2754.25	0.86	0.562	0.609	<	2.78	1.92	3.57	1.29
2934.71	0.43	0.562	0.888	0.000978	2.59	2.16	1.75	0.77
4353.46	0.3	0.562	0.145	1.02E-05	0.87	1.17	1.14	0.86
2324.54	0.99	0.597	0.358	<	3.03	2.04	4.32	2.29
1759.14	0.23	0.605	0.747	<	1.72	1.95	0.85	1.04
3625.92	0.34	0.674	0.342	<	2.29	2.63	0.81	2.63
6809.07	0.04	0.674	0.433	0.124	0.27	0.31	0.19	0.14
2379.9	0.63	0.709	0.216	<	2.6	3.24	3.31	3.19
2835.38	0.17	0.783	0.597	0.0653	2.69	2.53	1.15	0.79
5077.28	0.12	0.783	0.756	0.00136	1.44	1.56	0.79	0.75
3845.6	0.21	0.789	0.747	0.0373	3.74	3.53	1.37	1.75
4938.32	0.14	0.789	0.216	0.000014	0.87	1.01	1.03	0.69
5195.99	0.05	0.789	0.51	0.000868	0.45	0.49	0.33	0.3
2628.65	0.56	0.852	0.235	<	3.83	3.27	5.73	2.89
4587.1	0.05	0.874	0.424	7.31E-05	1.08	1.03	0.57	0.28
3823.59	0.79	0.882	0.797	0.201	13.97	13.19	9	7.88
4627.04	0.05	0.882	0.367	2.06E-05	0.73	0.79	0.66	0.46
5937.66	0.05	0.893	0.255	0.000014	0.48	0.43	0.71	0.31
8481.77	0.01	0.908	0.216	0.000224	0.18	0.17	0.2	0.07
2022.25	0.15	0.95	0.485	<	3.04	2.9	3.74	2.55
1466.47	0.14	0.95	0.747	<	3.22	3.07	3.19	3.46
3275.94	0.16	0.956	0.747	0.0289	9.11	9.26	4.16	6.19
5005.02	0.1	0.956	0.35	<	2.47	2.57	2.99	2.12
4964.86	0.02	0.998	0.304	<	2.88	2.86	3.58	2.47
3443.65	0.02	0.998	0.727	0.0803	7.12	7.1	1.7	2.28
4607.81	0	0.999	0.51	0.00538	0.87	0.87	0.53	0.31

Table SM 19. The discriminant masses between Poor Outcome SAH (PO-SAH) and Good Outcome SAH (GO-SAH) for cerebrospinal fluid samples collected at day 10 of SAH incident

Genetic Algorithm (GA)			Quick Classifier (QC)			Supervised Neural Network (SNN)		
**Mass (m/z)**	*Start Mass*	*End mass*	**Mass (m/z)**	Start Mass	End mass	**Mass (m/z)**	Start Mass	End mass
3700.26	*3691.38*	*3715.26*	1345.46	*1339.63*	*1348.93*	3700.26	*3691.38*	*3715.26*
2912.68	*2902.53*	*2927.42*	2428.48	*2418.12*	*2434.75*	3328.66	*3321.04*	*3341.59*
3275.94	*3265.8*	*3290.55*				2912.68	*2902.53*	*2927.42*
3743.41	*3729.67*	*3758.36*				3783.38	*3773.52*	*3795.88*
3389.93	*3382.45*	*3405.31*				3764.71	*3758.36*	*3773.52*
3783.38	*3773.52*	*3795.88*				3965.94	*3957.68*	*3976.91*
1749.32	*1746.2*	*1754.54*				1266.61	*1257.77*	*1272.98*
3557.31	*3549.05*	*3570.52*				7829.03	*7797.22*	*7837.84*
3538.47	*3531.33*	*3549.05*				3389.93	*3382.45*	*3405.31*
4567.44	*4557.03*	*4577.18*				3296.48	*3290.55*	*3306.82*
						3370.84	*3361.99*	*3381.4*
						1749.32	*1746.2*	*1754.54*
						1738.67	*1734.55*	*1743.17*
						4587.1	*4577.92*	*4596.15*
